# Recent nanotheranostic approaches in cancer research

**DOI:** 10.1007/s10238-023-01262-3

**Published:** 2024-01-19

**Authors:** Deepshikha Gupta, Priyanka Roy, Rishabh Sharma, Richa Kasana, Pragati Rathore, Tejendra Kumar Gupta

**Affiliations:** 1 Department of Chemistry, Amity Institute of Applied Sciences, Amity University, Sector-125, Noida, Uttar Pradesh 201313 India; 2 Department of Chemistry, Jamia Hamdard University, New Delhi, 110062 India

**Keywords:** Nanotheranostics, Cancer, Molecular imaging, Diagnostics, AuNP

## Abstract

Humanity is suffering from cancer which has become a root cause of untimely deaths of individuals around the globe in the recent past. Nanotheranostics integrates therapeutics and diagnostics to monitor treatment response and enhance drug efficacy and safety. We hereby propose to discuss all recent cancer imaging and diagnostic tools, the mechanism of targeting tumor cells, and current nanotheranostic platforms available for cancer. This review discusses various nanotheranostic agents and novel molecular imaging tools like MRI, CT, PET, SPEC, and PAT used for cancer diagnostics. Emphasis is given to gold nanoparticles, silica, liposomes, dendrimers, and metal-based agents. We also highlight the mechanism of targeting the tumor cells, and the limitations of different nanotheranostic agents in the field of research for cancer treatment. Due to the complexity in this area, multifunctional and hybrid nanoparticles functionalized with targeted moieties or anti-cancer drugs show the best feature for theranostics that enables them to work on carrying and delivering active materials to the desired area of the requirement for early detection and diagnosis. Non-invasive imaging techniques have a specificity of receptor binding and internalization processes of the nanosystems within the cancer cells. Nanotheranostics may provide the appropriate medicine at the appropriate dose to the appropriate patient at the appropriate time.

**Graphical abstract:**

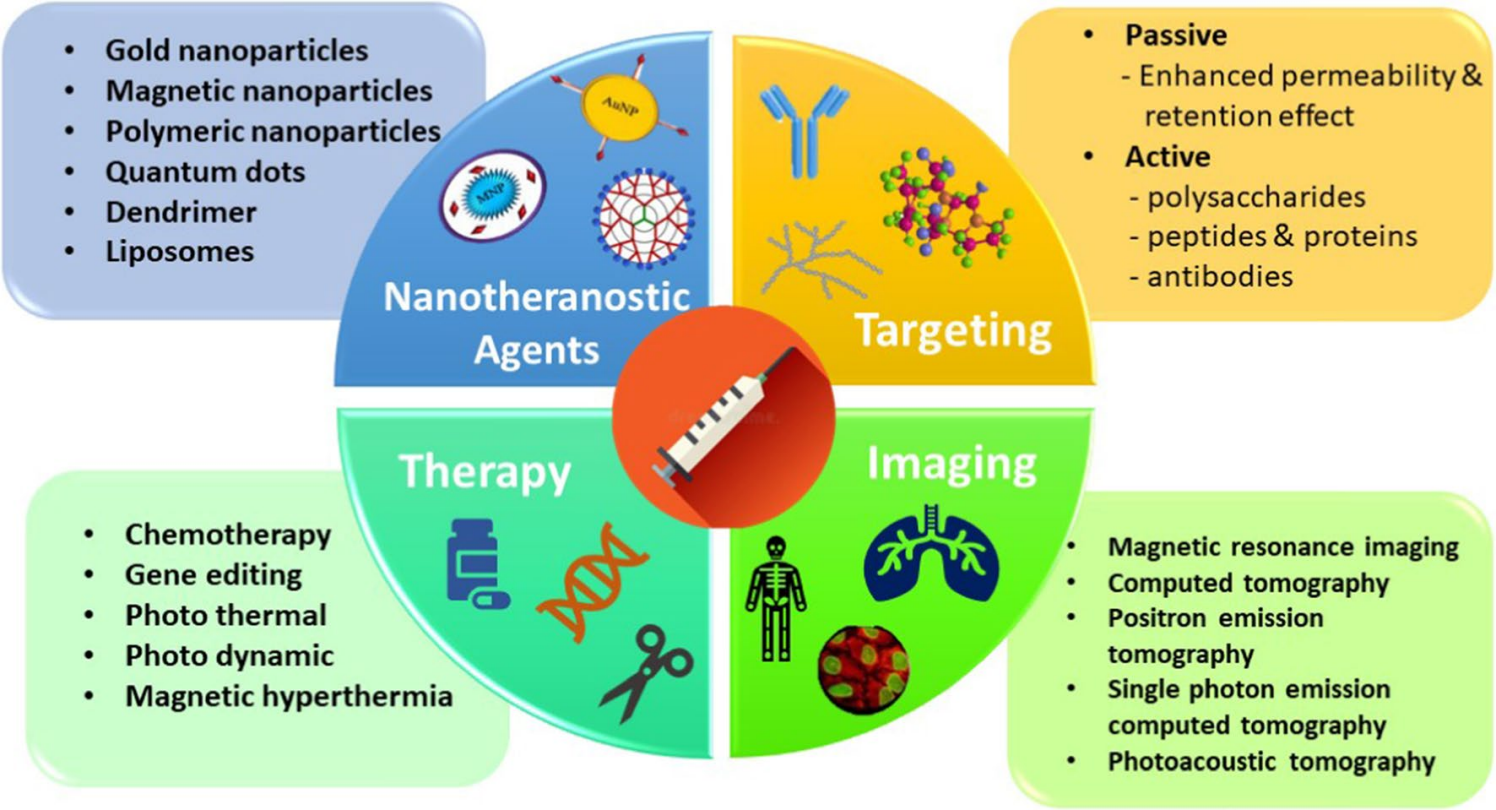

## Introduction

**Fig. 1:**
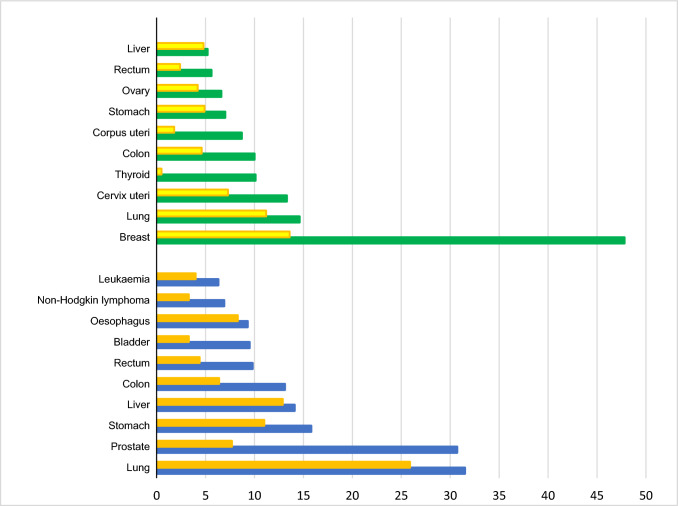
Estimated age-standardized cancer incidence (in male-blue bar, in female-green bar) and mortality rates (in male-orange bar, in female-yellow bar) (World) in 2020, all ages. Data source: International Agency for Research on Cancer (IARC), WHO, https://gco.iarc.fr/, GLOBOCAN2020. X-axis depicts the world population per 100,000, Y-axis depicts the types of cancer occurrences

**Fig. 2:**
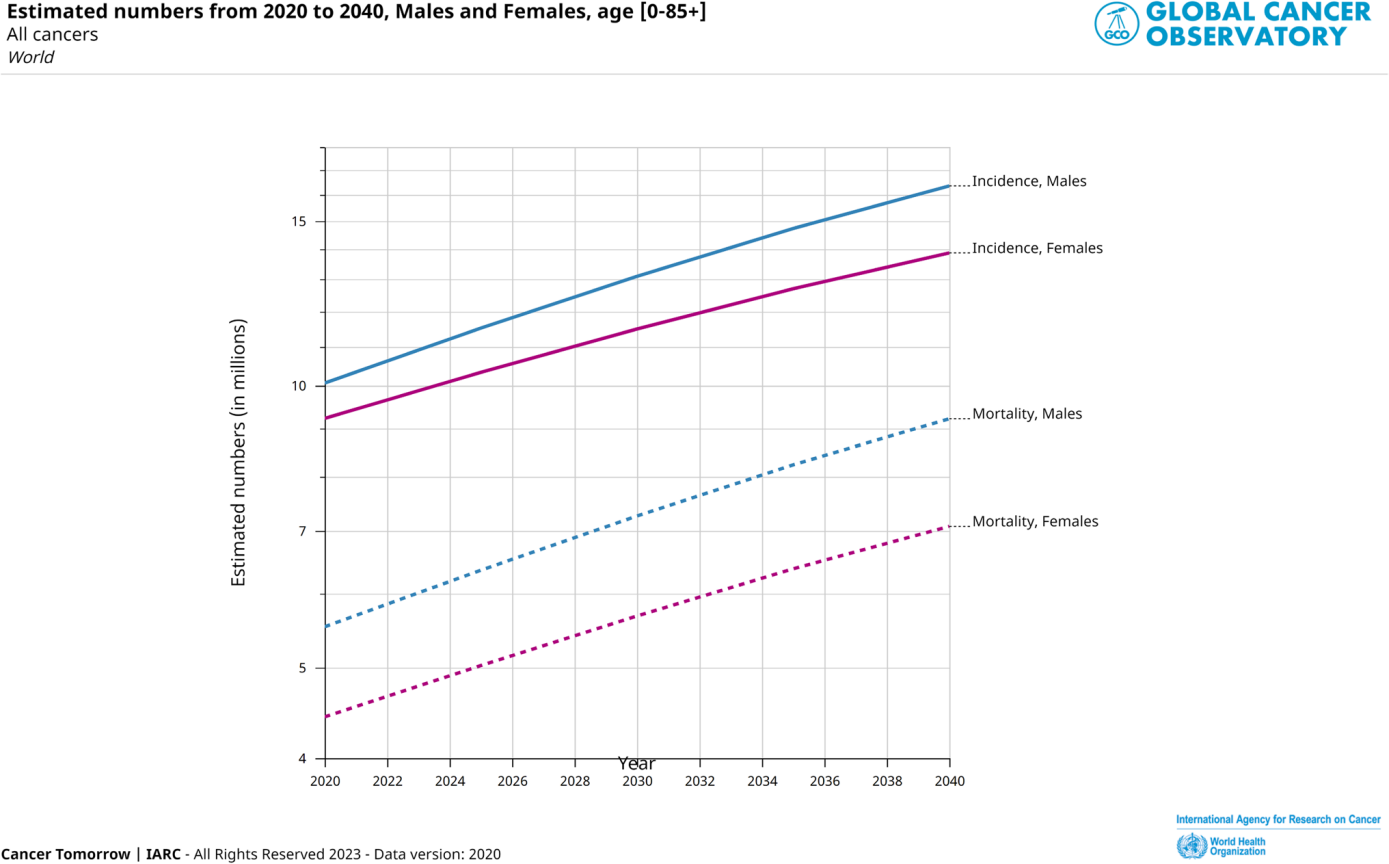
Estimated increase in numbers of cancer cases in males and females from 2020 to 2040, age [0–85+]. Data source: International Agency for Research on Cancer (IARC), WHO, https://gco.iarc.fr/, GLOBOCAN2020

Cancer is a serious and often life-threatening disease characterized by the uncontrolled growth and spread of abnormal cells in the body. It can have a profound impact on individuals and their families, as it can be physically, emotionally, and financially challenging. The second most common cause of death after a heart attack is cancer despite the great breadth of knowledge and information about it and improvements in its treatment. According to the International Agency for Research on Cancer (IARC), there were 19.3 million new cancer cases reported, and 9.9 million people died from cancer in 2020. In a study carried out by Zaorsky et al. [1] in the year 2016 based on US death certificate data from years 1973–2012, index-cancer, non-index-cancer, and noncancer causes for deaths of cancer patients were categorized. The group of people with malignancies of the testis, kidney, bladder, endometrial, breast, cervix, prostate, ovary, anus, colorectum, melanoma, and lymphoma saw the highest relative drop in index-cancer death (usually from > 60% to 30%). Patients with malignancies of the liver, pancreatic, esophagus, lung, and brain showed stable index-cancer deaths (usually > 40%). Patients with malignancies of the colorectum, bladder, kidney, endometrial, breast, prostate, and testis had the greatest noncancer causes of mortality; > 40% of fatalities were due to heart disease. As per the detail given by IARC, the estimated increase in the new cases from 2020 to 2025, and 2020 to 2040 would be 21.6 million, and 28.9 million, respectively (considering the age of the people from 0 to 85+). Figure 1 represents the reported types of cancer incidences and mortality in both sexes across the world in the year 2020 pointing that the most prevalent cancer type in females is breast cancer while in males lung and prostate cancer [2]. Estimated increase in cancer cases of all types from 2020 to 2040 in both sexes is depicted in Fig. 2. Cancer typically does not exhibit clinical symptoms in the early stages of the disease, and by the time it is discovered, metastatic lesions have already spread throughout the body, turning the disease from localized to systemic and becoming the primary factor in patient death [3].

The term "theranostics" refers to a treatment approach that integrates therapeutics and diagnostics to monitor treatment response and enhance drug efficacy and safety. Theranostics is a crucial component of personalized medicine that calls for predictive medicine advancements. The main aim is to ensure that this therapy is being provided to everyone in the form of customizable medicine, even though it will successfully gain importance to only a few patients. The use of both treatment and diagnosis and converting it into a single medicine is highly effective and useful only after certain clinical tests which shows positive test results and thus helps in the treatment of the disease by targeting those specific cancer-causing cells. This process is done in only one step with the proper approach [4]. The widespread use of nanomaterials remains challenging because of specific limitations like non-targeted distribution that result in low signal-to-noise ratio for diagnostics, complex fabrication, reduced biocompatibility, decreased photostability, and systemic toxicity of nanomaterials within the body [5, 6]. A relatively young but thriving area called nanotheranostics combines diagnosis and therapy to provide patients with individualized care. Scientists have successfully created smart nanoparticles for both diagnostics and therapies at the same time in vivo by combining nanomaterials from various sources, such as polymers, liposomes, micelles, and antibodies. Nanotheranostics will soon offer a realistic early stage treatment for cancer and other deadly diseases [7].

The emergence of nanoparticles in biomedical applications and customized medicine has made it possible to combine diagnostics and treatments into a single nanomaterial, known as "nanotheranostic". Nanotheranostic agents can be created by modifying nanoparticles to acquire theranostic characteristics. These nanocomposites can be customized for targeted medication delivery and are valuable instruments for eliminating cancer cells while concurrently evaluating the medicine's efficacy. In order to identify the "route and reach" of the medications and coordinate cancer treatment, nanotheranostic agents have become a wise strategy [8]. A field that combines nanotechnology, chemistry, materials science, and synthetic biology is called theranostic nanomedicine. The conceptualization of targeted drug delivery has the potential of concentrating on the consequences of the agents that are used for cancer-causing cells and making sure to keep them from any kind of toxicity which can affect the healthy tissues. The idea behind decreasing the side effects of targeted drug delivery whether it is in low-level or high-level drugs must concentrate on the cancerous site [9]. The composition of the drug delivery in nanotechnology is making it popular for cancer therapy. They are using the two main materials i.e., nanoparticles acting as a carrier agent and chemo drug. The drug can be used in different forms with nanoparticles either it can be in dissolved form or disseminated [10]. The main benefit of nanoparticles is that it increases the concentration of drug in larger amount to the target site of tissue with good biocompatibility and recognition which enhance the selectivity and ultimately it strengthens the medicinal reaction in nanotheranostics [11]. Integrated diagnosis and therapy systems that can offer effective cancer therapy are in high demand for personalized medicine. Targeted carrier systems with integrated imaging, therapeutic, and diagnostic modalities are being developed by scientists.

## Molecular imaging methods for targeted drug delivery

Molecular imaging methods are an essential tool for targeted drug delivery in cancer diagnostics by visualizing the molecular and cellular processes involved in progression of cancer. These techniques can detect location, size, and extent of cancerous cells and tissues, which can help in the development of personalized treatment plans and reduce the effect of it to some extent. Nanotheranostic is combination of both diagnostic and treatment which make it into single step process [12]. Targeted therapy for cancer cells using molecular targets, such as signal transduction inhibitors (STIs), gene regulation modulators, inhibitors of new blood cell growth, and hormonal treatments, has demonstrated improved properties in clinical applications. In contrast, traditional chemotherapy has limitations in detecting and treating specific cancer type for example, gastrointestinal tumor can be detected by zwitterionic nanoparticles or H-dots, which are precisely transported to the site of abnormal cell growth using IR rays [13, 14]. Nanotheranostic consisting of different types of contrast agents like radionuclide, fluorescent dyes, and gadolinium are used in number of molecular imaging methods which include X-rays, CT, MRI, SPECT, PET that can keep a track of pathological and physiological process which takes place in the body [15]. In the upcoming section we are described the various methods used for cancer imaging with examples.

### Magnetic resonance imaging (MRI)

MRI is one of the most extensively used non-invasive imaging technique. The detailed cross sectional image of soft and hard body tissues can be obtained by use of magnetic fields and radio waves that provide information about the size, location, and spread of cancer cells and tissues. MRI is appropriate for soft tissue as it is having good dimensions and resolution power for detection purpose, but it has limited sensitivity and a prolonged imaging duration. This is the reason why contrast agents have been developed to improves the signal power. The materials which are used in MRI are based for magnetic properties which includes paramagnetic, ferromagnetic, and superparamagnetic iron oxide (SPIONs) based agents [16].

Ultrasmall superparamagnetic iron oxide (USPIONs) which consist of ferumoxytol minimize the relaxation time of the MRI process. It generally reduces the metastasis in the liver as it has great therapeutic effect after being transporting to the targeted area of the body [17]. The usage of gold nanoparticles (AuNPs) is preferred as it has low cytotoxicity effect in comparison with SPIONs and when it is exposed to near infrared radiation (NIR) it generates a lot of heat which makes it appropriate for therapy of cancer, i.e., photothermal therapy (PTT). The most commercialized contrast agent is gadolinium (Gd), but, because of its small molecular weight, it has a limited lifespan in blood flow [18]. Gd-DTPA (diethylenetriaminepentaacetic acid)-polylysine, a macromolecular MRI contrast agent have slow blood clearance rate due to high molecular weight and produces a prolonged, almost constant tissue signal enhancement for the 60-min observation period [19].

The contrast agents which are related to manganese based inorganic compounds are not considered appropriate because of its high cytotoxicity and slow blood circulation except MnO nanoparticles that have low levels of cytotoxicity. It has also been studied that MRI play a significant role in diagnosing breast cancer, and P2-bMRI (predictive and prognostic) has been developed as cost-effective method for in vivo clinical examination of the tumor that can be customized for better accuracy in diagnosis and prognosis [20].

To create a dual-mode imaging contrast agent, NPs with composite cores made of bismuth and iron oxide were prepared. Bismuth serves as X-ray attenuating agent for CT, iron oxide provided contrast for MRI. Although the MRI contrast ability of the NPs was reduced as a result of the addition of bismuth to the iron oxide core, *in vivo* MRI and CT scans still demonstrated outstanding contrast from the composite NPs in both imaging modalities [21]. The types of contrast agents used for MRI and their significance are given in Table 1.

**Table 1:** Types of contract agents used for MRI with their significance

Type of contrast agent	Examples	Significance	References
Gadolinium-Based Contrast Agents (GBCAs)	Gadobutrol, Gadopentetate dimeglumine, Gadoterate meglumine, Gadobenate dimeglumine	Have enhanced contrast between healthy and abnormal tissues in MRI scans. GBCAs are generally safe but expensive and extensive imaging time needed	[23–25] [26]
Iron oxide nanoparticles	Ferumoxytol, Ferucarbotran	Used as a contrast agent for MRI scans of the liver, spleen, lymph nodes, and tumors. They are also used for imaging inflammation and cellular tracking in preclinical and clinical studies. Iron oxide nanoparticles are safe and well-tolerated, but they may cause mild adverse reactions, such as transient hypotension, flushing, or dyspnea	[17, 27]
Ultrasmall superparamagnetic iron oxide (USPIO)	Ferumoxides, Ferumoxtran-10	Used to enhance the contrast in MRI scans of the liver, spleen, lymph nodes, and tumors. They are also used for imaging inflammation, atherosclerosis, and cell trafficking in preclinical and clinical studies. USPIOs are safe and well-tolerated, but they may cause mild adverse reactions, such as transient hypotension, flushing, or dyspnea	[28, 29]
Perfluorocarbon-based Contrast Agents	Perflubron, PFOB	Used to enhance the contrast in MRI scans of the lungs, blood vessels, and tumors. They are also used for imaging inflammation and cellular trafficking in preclinical and clinical studies	[30]
Manganese-based Contrast Agents	MnDPDP, MnCl_2,_ Mn-MOFs	Used to enhance the contrast in MRI scans of the liver, pancreas, heart, and brain. They are also used for imaging cell metabolism, brain function, and neurodegenerative diseases in preclinical and clinical studies. Manganese-based contrast agents are generally safe and well-tolerated, but they may cause mild adverse reactions, such as nausea, dizziness, or flushing	[31, 32]
Hyperpolarized Gases	Hyperpolarized helium-3 (He-3), hyperpolarized xenon-129 (Xe-129)	These gases are used as contrast agents in lung MRI to visualize the airspaces and lung function in real-time. They have high sensitivity and specificity for detecting lung diseases, such as asthma, COPD, and pulmonary fibrosis. However, their production and administration require specialized equipment and expertise	[33, 34]
Metal–Organic Framework-based Contrast Agents	Zirconium-based MOFs, Gadolinium-based MOFs	These MOFs are a new class of contrast agents that have high stability, biocompatibility, and tunable properties. They can be functionalized with various targeting ligands and imaging probes to enhance their specificity and sensitivity. They are also suitable for multi-modal imaging and theranostics	[35, 36]
CEST Contrast Agents	Iopamidol-CEST, creatine-CEST	These contrast agents use chemical exchange saturation transfer (CEST) to detect and quantify specific molecules or metabolic processes in tissues. They have high sensitivity and specificity for imaging tumors, stroke, and neurodegenerative diseases. They also have potential for monitoring drug delivery and response	[37–39]

Additionally, MRI-based imaging (T1, T2, triple and quadruple multi-modal imaging) can be clubbed with therapy (chemo-, thermal-, gene- and combination therapy), connecting a range of topics including hybrid treatment options, novel targeting strategies, and tumor microenvironment-responsive drug release (*, e.g.,* redox and pH-responsive nanomaterials) [22].

### Computed tomography (CT)

X-rays are used in CT scans to provide finely detailed pictures of the internal organs that can determine the size, location, and spread of cancer cells and tissues. CT has several benefits which includes maximum resolution power, intense penetration power and the convenience of 3-D tissue regeneration but is not able to distinguish between the tissues which have same physical property. To minimize it, contrast agent (tungsten and iodine compounds) and the organic based materials which consist of Au nanoparticles and high atomic number element is used to increase the effectiveness of the CT imaging [40].

A new type of polymer-based nanoparticles, containing iodine and coated with polyethylene glycol (PEG), has been introduced as a contrast agent for targeting cancer cells. These nanoparticles have shown promise in their ability to improve the imaging and detection of cancer-causing cells [41]. CT is constantly used with other anatomic imaging techniques like as MRI, Positron tomography, and molecular imaging to improve operational imaging and reflect basic anatomical transformation by deploying new signals that detect cellular activities [42].

Research indicates that the binary contrast agents consisting of barium (Ba^2+^) and holmium (Ho^3+^) combined with hydroxyapatite, a vital component of bones and teeth, have been developed [43]. Such type of contrast agent was found beneficial for examining cancer cells in breast [44].

According to a study by Aydogan et al. [45] the use of 2-DG (2-deoxy-D-Glucose) labeled AuNPs was used as X-ray computer tomography contrast agent for *in vitro* cellular uptake of human alveolar epithelial cancer cell line, A-549. Furthermore, the amalgamation of both PET and CT shows effective functionality and provides detailed facts based on molecularity. F18 (fluorodeoxyglucose) as a contrast agent have shown effective properties in detecting thyroid cancer but CT imaging alone is not that efficient which showed only 80% effectiveness compared to PET and CT together with 84% effectiveness in diagnosing the cancer [46].

Contrast-enhanced computed tomography (CECT) also helps in determining the renal cell carcinomas (RCCs) due to which malignant and benign neoplasm start developing abnormally in the body, this will eventually help in gaining information regarding differential diagnosis and prognosis of RCCs [47].

### Positron emission tomography (PET)

This technique involves the use of radiolabeled tracers that bind to specific molecules expressed by cancer cells. PET scans can provide information about the metabolic activity of cancer cells and can help in the detection of cancer at an early stage. Although PET holds excellent sensitivity, infinite depth of penetration, and quantifiable capabilities, it emerges as a strong technique for cancer detection and functional imaging of cancer cells and other disorders. The combination of MRI/CT or PET/CT is called as dual imaging method. These devices will ultimately help in gaining correct information related to molecularity of the human body [42].

The most used biomarkers are FDG or F18 (18-fluorodeoxyglucose) PET/CT. One of the leading causes of cancer-related fatalities in this generation is hepatocellular carcinoma (HCC). To detect HCC, therapeutic intervention is performed followed by the application of radiofrequency with 18F-FDG to assess the therapy through PET/CT and determine the benefits of using metabolic parameters. A comparison between diffused MRI and PET/CT was conducted to observe the outcomes, which revealed that diffusion weighted-MRI was more effective than PET/CT in diagnosing tumor cells [46].

The metabolic properties FDG PET/CT has a nature of predicting the viability rate of the patient who is suffering from head and neck cancer. The head and neck squamous cell carcinoma basically depend upon the denseness of lymphocyte that infiltrate tumors (TILs) which has the ability to act as a biomarker present in the microenvironment [48]. Prostate specific membrane antigen (PSMA) PET/CT has significantly progressed in prostate tumor treatment in current history. In males with clinically diagnosed prostate cancer, PSMA PET/CT had a greater sensitivity of nearly 90% in detecting pelvic lymphadenopathy abnormalities compared to traditional tomography. There has also been an increase in the identification of lymph node metastases. PSMA PET/CT has also been demonstrated to be less expensive than standard scanning; hence, it may be assumed that PSMA PET/CT will be more economic than traditional image analysis. According to a study by Sar et al., the introduction of [^68^ Ga]GaPSMA-11 PET/CT to examine its predictive performance and found that it can aid in the early diagnosis of prostate cancer and is a cost-effective approach [49].

The diagnosis of urokinase receptor present in breast and urinary tract which can be examined by Ga PET (^68^ Ga-NOTA-AE105) a type of radio-ligand. This is being studied on individuals with the afore mentioned malignancies, and no adverse effects have been recorded concurrent treatment and scanning by PET/CT [50].

According to McDonagh et al. a more efficient and promising method of producing a multiple coated nanoparticles containing cerium oxide and an efficient internal core labeling of (CONPs) with the therapeutic PET radioisotope, Zr-89, enabling for comprehensive PET scanning as it showed increase in the renal clarity rate and therapeutic efficacy [51].

### Single photon emission computed tomography (SPECT)

SPECT involves the injection of a radioactive tracer into the patient's bloodstream. In the targeted tissue, the tracer builds up and emits gamma rays that may be seen by a specialized camera. By moving the camera around the patient, several 2D pictures of the desired tissue are created from various perspectives, and these photos are used to create SPECT images. A 3D picture of the tissue under study is subsequently created by combining these images using computer techniques. The location and concentration of the tracer in the tissue are visible in the ensuing picture.

In cancer detection and imaging, SPECT can be used to identify cancerous cells, determine the size and location of tumors, and evaluate the effectiveness of cancer treatment. It is particularly useful in detecting and staging certain types of cancers, such as bone cancer and thyroid cancer. SPECT can also be used to guide biopsies and other surgical procedures. One advantage of SPECT is that it is a non-invasive procedure that does not require surgery or anesthesia. Additionally, the radioactive tracer used in SPECT is generally well-tolerated by patients and has few side effects. According to the use of SPECT, it was found bone metastasis can be detected by introducing the ^99m^Tc MDP (methylene diphosphonate) at the specific place of bone via injecting it to the blood [52]. Fluorescent dyes or bioluminescent proteins that bind to specific molecules expressed by cancer cells. Optical imaging can provide real-time images of cancer cells and tissues and can help in the detection of cancer at an early stage. Even breast cancer is the leading type of cancer in women those accounts for 60% of all metastatic bone cancer in patients. It has a significant influence on the quality of sustenance of life of women with breast cancer. Inflammation, pathological cracks, spinal stenosis, and hypokalemia of malignancies are clinical consequences of bone metastasis [53].

The use of radioactively labeled biomolecules to target receptors is an effective approach for the early identification of breast cancer. Creating a high-sensitivity tool for detecting metastatic breast cancer using SPECT and PET has become crucial. Several studies have identified that the activation of a particular receptor plays a significant role in the onset of breast cancer [54].

The most used contrast agent besides ^99m^Tc is Indium labeled liposomes and micro colloids. SPECT has a many advantages like high responsive and penetrating power but it does not provide any anatomical knowledge and have poor resolution ability [55]. However, there are some limitations to SPECT technique. It may not be as accurate as other imaging techniques, such as MRI or CT, in detecting small tumors or lesions. Additionally, the use of a radioactive tracer, can pose a risk of radiation exposure to both the patient and the medical staff and appropriate safety precautions should be taken. It is a non-invasive and well-tolerated procedure that can provide important information about the location and concentration of cancerous cells, helping doctors to diagnose and treat cancer more effectively.

### Photoacoustic tomography (PAT)

Photoacoustic tomography (PAT) is a non-invasive imaging technique that combines the advantages of optical and ultrasound imaging to visualize the tissue structures and functions *in vivo.* When biological tissues are exposed to a laser beam during PAT that causes ultrasonic waves to be produced. These ultrasonic waves are then detected by an ultrasound transducer, which is used to reconstruct the images of the tissue.

PAT has shown great potential for cancer diagnosis and monitoring. Cancer cells have higher levels of certain molecules, such as oxygenated and deoxygenated hemoglobin, melanin, and lipids which have strong absorption of light at specific wavelengths. By using PAT, these molecules can be targeted, and the resulting photoacoustic signals can be used to identify cancerous tissue. PAT can also be used to monitor the response of tumors to treatment. By repeatedly imaging a tumor using PAT, the changes in the photoacoustic signals can be tracked to determine the effectiveness of the treatment. One advantage of PAT over other imaging techniques such as CT and MRI are its ability to provide functional information about the tissue. PAT can provide information about the oxygenation of blood vessels, the distribution of specific molecules, and the metabolic activity of tissue.

AuNPs can also be applied in photoacoustic tomography (PAT), as contrast agent when excited at the wavelength of their resonance, undergo photothermal conversion, which results in the creation of acoustic waves (owing to thermoelastic expansion) that may be detected by an ultrasound transducer. Gold nanorods are extremely useful for PAT because of their cylindrical form, which exhibits distinctive SPR toward the NIR. The use of reporter-gene products has also enhanced PAT molecular imaging in deep tissue [29, 56]. Reporter genes products are proteins encoded by reporter genes that are used to monitor and measure gene expression or other cellular processes. These genes are artificially introduced into cells or organisms to provide a convenient and measurable readout of specific cellular activities. Silver, palladium and platinum nanoparticles can also be used to enhance contrast and sensitivity in PAT for cancer detection due to their strong optical absorption properties and photoacoustic signal generation. Silver NPs undergo dissolution in biological system over the period of time making them more suitable as compared to gold NPs [57]. Homan et al. investigated the potential of porous silver layer (for release of therapeutic agents) deposited on the surface of spherical silica cores (180 to 520 nm diameter) as non-toxic imaging agent upto the concentrations of silver up to 2 mg/ml [58]. The same group in another experiment checked the directional antibody conjugation to the silver nanoplate surface as proof of molecular sensitivity with pancreatic cancer cells in both in vitro and in vivo models as non-toxic imaging agent upto 1 mg/ml concentration [59]. Coating of Au nanorods with silver decreased their photoacoustic signal but upon etching with ferricyanide solution (conc. 1 mM) oxidized the silver into Ag^+^ ions which were released to show excellent bactericidal efficacy for both gram positive and gram-negative strains. It showed 730% increase in photoacoustic signal post etching [60]. Similar to silver, platinum and palladium nanoparticles can be functionalized with targeting ligands or antibodies. This allows for active targeting of specific cancer cell markers, further enhancing the specificity of cancer detection by concentrating nanoparticles in tumor regions. Non-invasive cancer theranostics using bimetallic hyaluronate-modified Au-platinum (HA-Au@Pt) nanoparticles enhanced the absorbance in the NIR region and broadened the absorption by the surface plasmon resonance (SPR) coupling effect in skin cancer photothermal therapy and imaging [61]. Palladium based nanomaterials showed high photothermal conversion efficiency and high photothermal stability over Au and Ag based nanoparticles [62].

## Current nanotheranostic platforms for cancer

Nanotheranostics and theranostic nanomedicine are cutting-edge strategies that provide an integrative approach connected to diagnostic and therapeutic properties in one single entity. In cancer research, it has potential to revolutionize cancer diagnosis, treatment, and management for which a variety of nano-carriers have been created including dendrimers, liposomes, polymer conjugations, and metal & silica nanoparticles (NPs). Future applications of nanotheranostics may include the non-invasive discovery of biomarkers, the treatment of cancer, and the delivery of drugs [63].

**Fig. 3:**
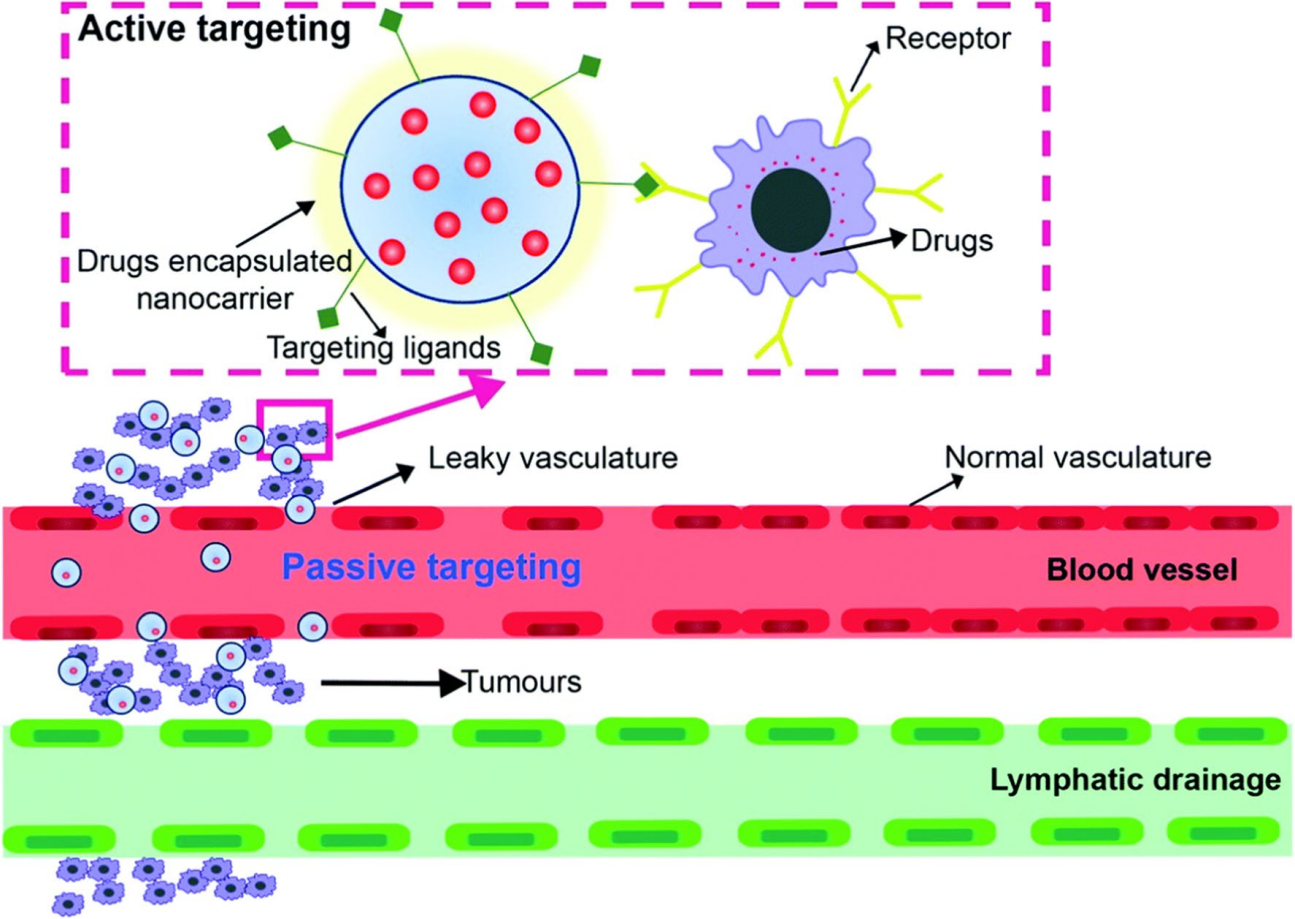
Mechanism of active and passive drug targeting by nanocarrier, Reproduced from Ref. [65] with permission from the Royal Society of Chemistry. This article is licensed under a Creative Commons Attribution-NonCommercial 3.0 Unported Licence

The malignant microenvironment is characterized by faulty vascular structures and structural abnormalities. Active and passive targeting are two strategies employed in drug delivery to enhance the specificity and effectiveness of therapeutic agents, particularly in the context of treating diseases like cancer. These strategies aim to improve the delivery of drugs to the desired target tissues while minimizing their exposure to healthy tissues, thereby increasing therapeutic efficacy, and reducing side effects. Passive targeting takes advantage of the physiological characteristics of the target tissue, such as its microenvironment and vasculature, to achieve selective drug accumulation. The primary mechanism for passive targeting is the Enhanced Permeability and Retention (EPR) effect [64]. This effect is particularly prominent in tumors, where abnormal and leaky blood vessels allow for preferential accumulation of nanoparticles and drugs within the tumor tissue. In passive targeting nanoparticles or drugs are designed to have specific physicochemical properties (e.g., size, surface charge) that allow them to evade rapid clearance from the bloodstream. These particles can extravasate (leak) from the blood vessels more easily in regions with leaky vasculature, like tumor tissues, due to the EPR effect. Once they accumulate in the target tissue, they release the drug payload over time, enhancing the therapeutic effect. For solid tumor cells the drug is delivered passively to the targeted cells. Therefore, the initial focus should be on meeting the essential requirements in the entire process of targeted drug delivery [65]. The important factor which needs to be followed is the appropriate size of the nanocarrier (10 to100 nm) for eliminating the chances of filtration through kidney and entrapment of nanocarrier in the liver. For effective avoidance of renal clearance, the nanocarrier charge must be anionic or neutral. Doxil® (doxorubicin hydrochloride liposome injection) is an example of a liposomal formulation used in the treatment of ovarian and breast cancer [66]. An albumin-bound nanoparticles for drug delivery, Abraxane® (paclitaxel protein-bound particles for injectable suspension) [67], can exploit the EPR effect to accumulate in the tumor tissue and have a longer circulation time in the blood compared to conventional paclitaxel. The active and passive targeting drug delivery mechanism is shown in Fig. 3.

Active targeting involves the use of ligands or molecules that can specifically recognize and bind to receptors or markers on the surface of target cells. This binding interaction allows for more precise drug delivery to the intended site. Active targeting uses ligands bound receptors that can bound to the surface of tumor cells for enhanced selectivity. After binding the drug, they are delivered to the specific cancer cell [65, 68]. This strategy is particularly useful when the target tissue doesn't exhibit strong EPR effects or when higher specificity is required. In active targeting ligands, such as antibodies-drug conjugates, peptides, aptamers, or small molecules, are attached to the surface of drug-loaded nanoparticles. These ligands are chosen based on their ability to selectively bind to receptors or markers overexpressed on the target cells. Once administered, the ligand-decorated nanoparticles circulate in the bloodstream until they encounter the target tissue, where they bind specifically to the cell surface. The drug is then internalized by the target cells via receptor-mediated endocytosis, leading to higher drug concentrations at the desired site. Peptides can be conjugated to drugs or nanoparticles to increase their selectivity for tumor cells. For example, the cyclic RGD peptide can target αvβ3 integrin, which is overexpressed on the surface of tumor cells, and has been used for drug delivery to tumor cells [69]. Combining both active and passive targeting can lead to even more effective drug delivery systems by maximizing accumulation at the target site while also ensuring specific interaction with target cells.

In the upcoming sections various metal-based (including magnetic NPs, gold NPs, and quantum dots,) silica-based, dendrimer-based, polymer-based, and liposome-based nanotheranostic agents are discussed in detail. Each platform has its advantages and limitations, and the choice of platform depends on the specific application. Our focus will be now on most important nanotheranostic platforms which are discussed in detail below:

### Silica-based nanotheranostic agent

Silica-based nanotheranostic agents have gained significant attention for their potential use in cancer diagnosis and therapy. When compared to conventional drug nano-carriers, silica nanoparticles exhibit substantial benefits due to their high surface area and customized mesoporous structure [70]. Mesoporous silica nanoparticle (MSNs) based systems also provide high surface area, different morphologies, adjustable pore size, functionalizable surface, and acceptable biological safety [71]. The targeting moieties enable these agents to selectively bind to cancer cells; while, the imaging agents allow for non-invasive detection and monitoring of the cancer. Functionalization of MSNs with different organic and inorganic molecules, polymers, surface attachment of other NPs, loading and entrapping cargo molecules with on-desire release capabilities, lead to seemingly endless prospects for designing advanced nano constructs exerting multiple functions, such as simultaneous cancer-targeting, imaging and therapy [72]. MSNs based imaging agents have gained extensive attentions in hepato cellular carcinoma detection, which can serve as a multifunctional nanoplatform with controllable size and facile surface functionalization [73].

The multifunctional platform based on fibrous silica nanoparticles enable targeted

“Triple Negative Breast Cancer theranosis”. Enhancement of in vivo targeted imaging and synergistic multi-modal therapy with concomitant toxicity reduction of treatment [74]. Cui et al. have reported the synthesis of gold nanoparticles-decorated fluorescent silicon nanorods featuring high photothermal conversion performance and good photothermal stability enable a total ablation of tumors and prolong the survival time of mice. Perfluorohexane (PFH) and superparamagnetic iron oxides (SPIOs) are the two materials that have been used in the development of multifunctional nanotheranostic agents for MRI-guided HIFU (high-intensity focused ultrasound) tumor ablation. Silica lipid is used as the carrier for the PFH and SPIOs, as it provides a stable platform for the materials to be loaded into. The resulting multifunctional nanotheranostic agent is known as silica-lipid-encapsulated perfluorohexane-superparamagnetic iron oxide nanoparticles (SSPN). PFH component of SSPN is a highly volatile liquid that vaporizes when exposed to heat, which is generated by HIFU. This vaporization causes a local increase in pressure and temperature, leading to the mechanical disruption of nearby cancer cells. This is known as cavitation, and it can be used to destroy tumor tissue. SPIOs in SSPN provide contrast for MRI imaging, allowing for real-time monitoring of the tumor ablation process. This enables real-time imaging and precise targeting of the HIFU beam and ensures that only the tumor tissue is destroyed, while surrounding healthy tissue is spared. It is temperature-sensitive because of its distinctive liquid-to-gas transition [75]. Along with the discovery of tumor heterogeneity and complexity, the use of diagnostics to direct or assist therapeutic methods has shown considerable promise [76].

For poorly soluble drugs, Tan et al. [77] suggested silica-lipid hybrid (SLH) microcapsules. Zhang et al. [78] has reported the biocompatible mesoporous silica-lipid bilayer nanoparticles with high drug loading, which could release doxorubicin (DOX) in response to hyperthermia and reduce premature release. In a study carried out by Prasad et al. [79] which describe the extremely high uptake and retention of carbanosilica, a mesoporous silica material containing embedded graphene quantum dots (GQDs), in solid tumors. It is a hybrid material composed of carbon and silicon, and it has unique physical and chemical properties that make it attractive for biomedical applications. Carbanosilica is non-toxic, inert, suitable for *in vivo* experiments due to biocompatible nature. It exhibits strong fluorescence, which makes it useful for imaging applications. It can be functionalized with drugs, making it a potential platform for targeted drug delivery. It can be functionalized with magnetic nanoparticles for MRI applications. After NIR light exposure, a brief (0.5 h) emission from the tumor area is seen, and with a single dose of nanohybrids, this emission is maintained for up to a week (tested up to 10 days). Carbanosilica is a potential nanotheranostic agent, showing 68.75% tumor shrinkage compared to without NIR light exposure (34.48%), thanks to its emissive and photothermally active GQDs and porous silica shell (around 31% drug loading).

### Dendrimer based nanotheranostic agents

**Fig. 4:**
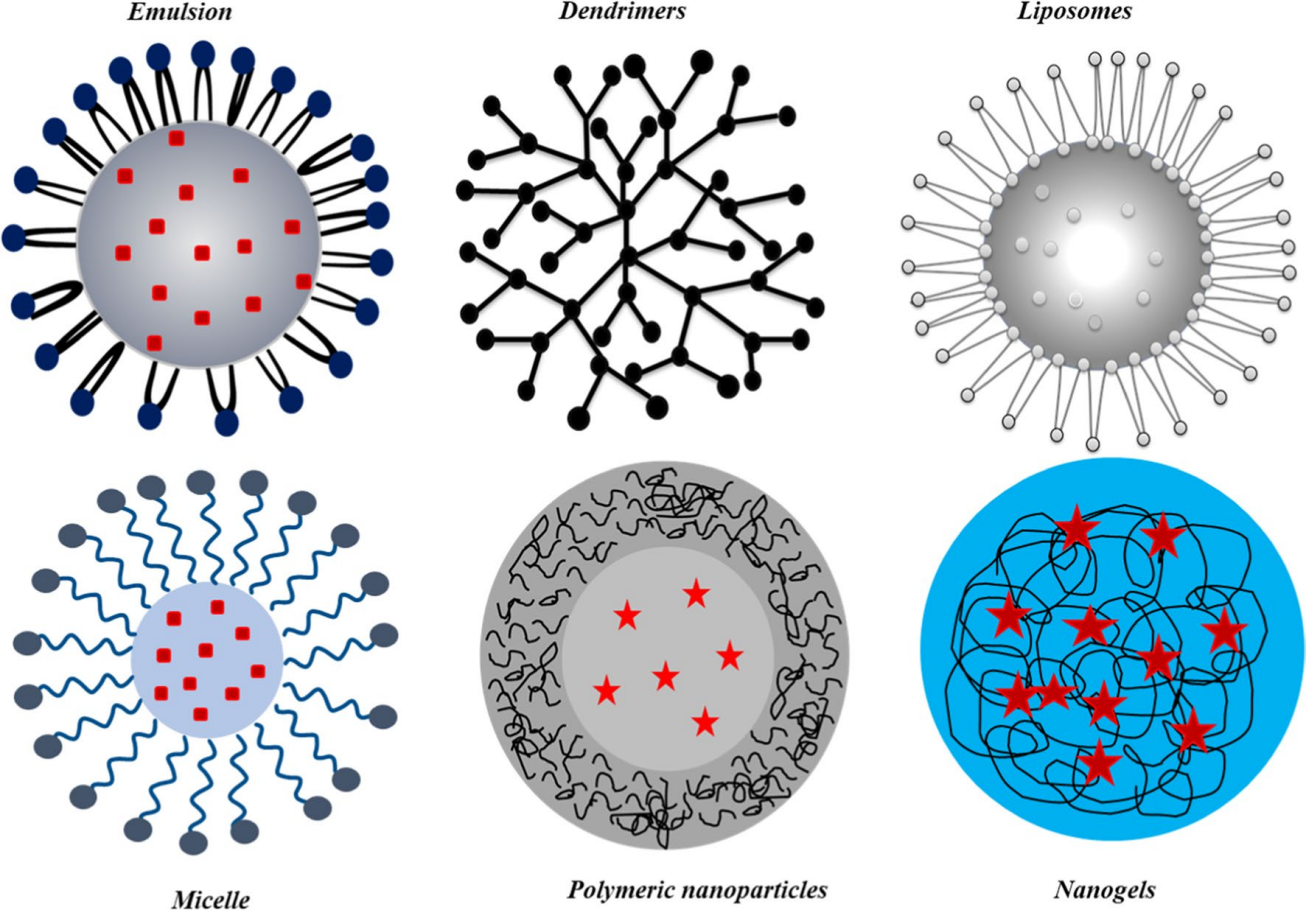
Different types of controlled drug delivery carriers: Emulsions, dendrimers, liposomes, micelle, polymeric nanoparticles, and Nanogels

Dendrimers are highly branched, nano-sized, and monodisperse polymers that can be synthesized with precise control over their size, shape, and surface chemistry. Due to their unique properties, dendrimers have attracted considerable attention as versatile platforms for the development of nanotheranostic agents for cancer imaging, diagnosis, and treatment. All the features of dendrimers exhibit a consistent pharmacokinetic behavior, which may ensure the desired biodistribution and effectiveness of these materials [80]. Dendrimers carry multiple imaging and therapeutic agents on their surface or within their internal cavities. This allows for the development of multifunctional nanotheranostic agents that can simultaneously target cancer cells, visualize tumor sites, and deliver therapeutic payloads. The inside of the dendrimer can be used to trap drug molecules; while, the peripheral functional groups can be used to build complexes and modify drug molecules through covalent interactions [81]. Dendrimers have thus been utilized in the biomedical field for *in vitro* diagnosis in gene therapy to transfer genes through the cell membrane, and in regenerative medicine [63, 82, 83]. Different kinds of controlled nano-carriers for drug delivery systems are shown in Fig. 4.

### Liposome-based theranostic agents

Liposomes can be explained as phospholipid vesicles which are made up of one or maybe more circular bilayers that enclose distinct aqueous regions. Liposomal technology has a unique capacity to entrap both lipophilic and hydrophilic molecules which allows them to incorporate a wide spectrum of medications. Water-insoluble compounds are introduced into the phospholipid bilayer; whereas, hydrophilic compounds can be confined in the aquatic core. Furthermore, the vast aquatic core and biocompatible lipid shell allow for the administration of a wide range of biomolecules, including DNA, proteins, and diagnostic agents [84].

The effectiveness of liposomes, along with current innovations in nanotechnology, has inspired the creation of a variety of innovative liposome-like nanomaterials with better drug delivery capabilities. There are five basic types of nanostructured materials: polymer-liposomes, nanoparticle liposomes, core–shell phospholipid polymer, naturally occurring membrane developed from vesicles, and coating of the membrane. These have got a lot of attention and have become prominent drug delivery systems [85].

**Fig. 5:**
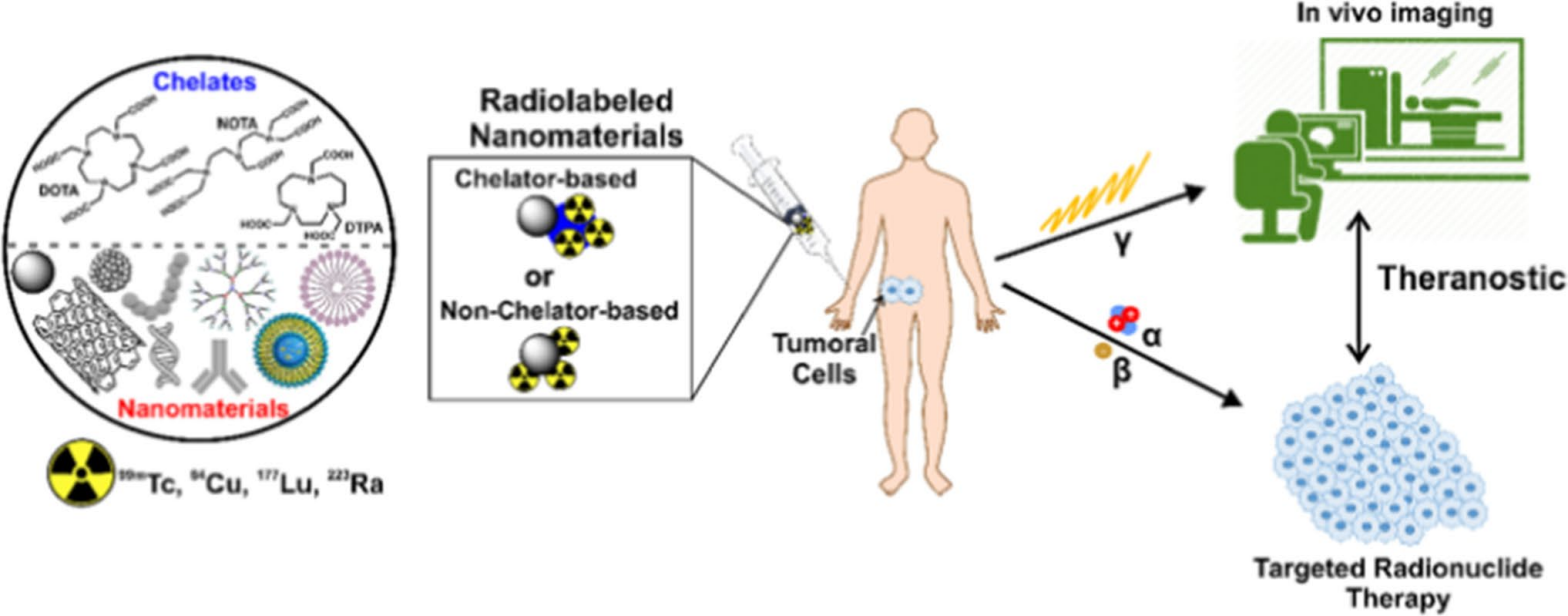
Radiolabelled nanomaterials such as ^99m^Tc, ^64^Cu, ^177^Lu, and ^223^Ra for molecular imaging and radionuclide therapy. Reprinted with the permission from [87] under http://creativecommons.org/licenses/by/4.0/

Customized liposomes can carry both medications and monitoring agents in tumor tissue, allowing for a therapeutic and diagnostic strategy with enormous potential in customized therapy. Liposome radiolabeling has been used in experimental investigations for so many years to evaluate liposome *in vivo* activity and has been an essential technique in the discovery of liposomal medications [86]. As shown in Fig. 5, the radionuclide used for labeling and loading liposomes for SPECT imaging are ^67^ Ga, ^111^In, ^123^I, ^99m^Tc while for PET imaging ^18^F, ^64^Cu, ^124^I [86].

### Metal-based nanotheranostic agents

Metal-based nanotheranostic agents have both diagnostic (MRI, PET, CT contrast agents) and therapeutic properties. They are made up of metal nanoparticles functionalized with targeting ligands and/or therapeutic molecules, such as drugs or nucleic acids [88]. Initially, MRI has only used metal-derived nanoparticles as contrast agents. But, nowadays, green chemical synthetic methods and multiple surface modifications and core changes have led to a wide range of biological uses for metal-derived nanoparticles, including tumor imaging, diagnostics, and treatment [89]. A developing field of investigation is focused on utilizing nanoparticles to enhance the efficacy of radiation-based cancer treatments, including proton therapy [90]. Nanoparticles such as gold, bismuth, tungsten, tantalum, ytterbium, gadolinium, silver, iron, platinum, and lead have been investigated by different group of scientists for various nanotheranostic applications [91]. We have discussed the use of gold nanoparticles and magnetic nanoparticles in various theranostic platforms in detail.

#### Gold nanoparticles (AuNPs)

**Fig. 6:**
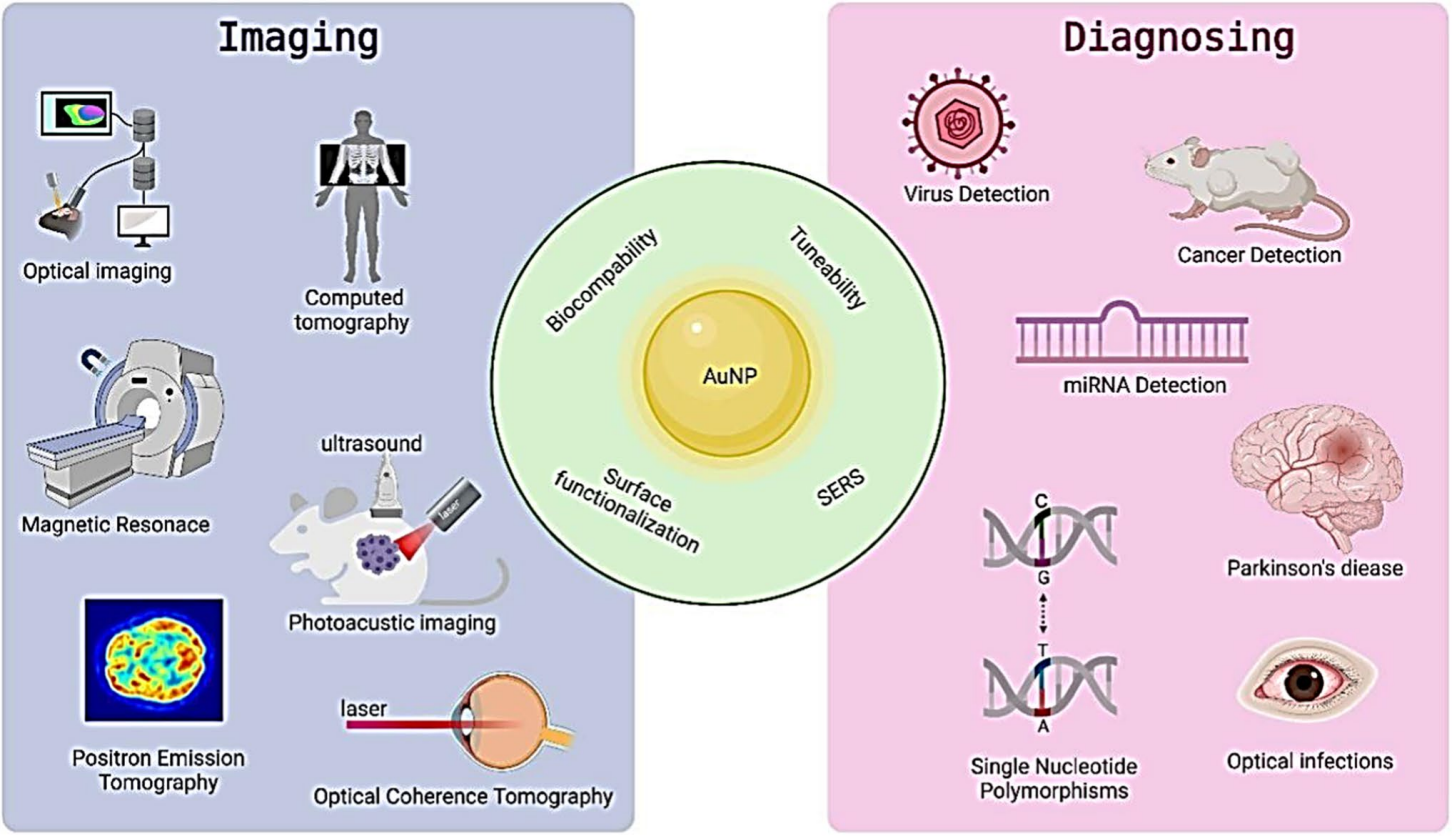
Properties and application of AuNPs in imaging and diagnosing. Reprinted from [94] an open access article under Creative Common CC BY license

Due to its high atomic number and density, and a high X-ray absorption coefficient, AuNPs are attractive CT contrast agent materials. AuNPs have unique optical properties, low *in vivo* toxicity, stability, which allow them to absorb and scatter light, making them ideal for imaging applications such as surface-enhanced Raman scattering (SERS) and photoacoustic imaging (PAI). AuNPs can also be functionalized with targeting ligands and therapeutic molecules, which make them useful for targeted drug delivery. Additionally, because of the enhanced permeability and retention (EPR) effect, they might accumulate at tumor areas, making them desirable for imaging diagnostics. X-ray attenuation coefficient and biocompatibility of AuNPs make them a viable CT contrast agent in comparison with conventional contrast agents that have many side effects. The ease with which they can be functionalized by virtually any type of (bio)molecule, and the fact that they can be made using straightforward and reliable synthetic processes have all made AuNPs effective theranostic tools. The in vivo mobility, biostability, circulation time, biodistribution, renal clearance, metabolism, toxicity, and catalytic activity of gold nanostructures are substantially influenced by the size and shape of nanoparticles. The size of nanoparticles has a significant impact on their optical characteristics and theranostic effectiveness. Larger AuNPs, or those measuring 34.8 nm in size, have higher X-ray attenuation properties than smaller ones. Others have demonstrated that smaller nanoparticles, such as those of sizes 4 nm and 13 nm, show better behavior than those of sizes 40 nm and 60 nm, respectively. Studies shows that, compared to nanocages and 45 nm spheres, 15 nm spherical particles are 1.5–2.4 times more effective in absorbing substances [92]. AuNPs with a spherical shape tend to be more stable and have higher cellular uptake compared to other shapes. On the other hand, AuNPs with non-spherical shapes, such as rods, stars, or triangles, can have unique optical properties and enhanced scattering, which can improve their imaging properties. Anisotropic gold nanostructures like nano-hexapods, nano-stars and nanorods have been shown to be superior photothermal agents to spherical nanoparticles. Compared to nano-hexapods, nanocages have a greater photothermal conversion efficiency per gold atom. However, a few contradictory investigations have shown that when exposed to an X-ray source, spherical nanoparticles kill more cells than nanorods and nanospikes do [93]. The localized SPR (LSPR), which is brought on by the activation of a collective and coherent oscillation of conduction electrons near the surface of AuNPs by an external laser of a particular wavelength. This oscillation produces scattering peaks, spectrum absorption, and local field enhancements because it is in resonance with the incident light frequency. AuNPs' scattering and absorption characteristics can be improved by adjusting their SPR. Water and hemoglobin, have lowest absorption coefficients and scatters the excitation emission photons in NIR. This allows for maximum laser penetration in the tissue and reduces autofluorescence of the non-laser components. AuNPs are ideal options for photothermal therapy and photoacoustic imaging agents due to their LSPR and NIR spectral range. The properties of AuNPs such as biocompatibility, tuneability, surface functionalization, etc., and their applications in imaging and diagnosis are shown diagrammatically in Fig. 6.

##### Cancer diagnosis, Imaging and therapy using Gold NPs

Gold nanoparticles have been investigated as drug carriers due to high drug loading capacity with improved hydrophilicity and stability of drugs, enhanced permeability and retention (EPR), and enhanced tumor selective accumulation by modifying surface with targeting ligands.

According to research, conjugated Au nanorods transported doxorubicin to tumors with an optimal tumor/muscle ratio of 16.6 at 5 h after injection. The biodistribution of nanorods could be clearly seen and measured by micro-PET/CT *in vivo* since the nanorods were labeled with radioactive ^64^Cu [29, 95].

Citrate-stabilized AuNPs coated with poly-L-lysine and rhodamine B isothiocyanate (RITC) allow for CT cell tracking both *in vitro* and *in vivo*, and they may have uses in CT image-guided interventions for the injection of cellular therapies [96]. A human lung cancer cell line underwent *in vitro* and *in vivo* CT imaging using acetylated dendrimer-entrapped gold nanoparticles (AuDENPs) (SPC-A1 cells). In xenograft tumor models, intratumoral and intraperitoneal delivery of acetylated AuDENPs enabled the X-ray identification of cancer cells. Acetylated AuDENPs are an excellent CT contrast agent because they are biocompatible and do not impair cell survival, according to *in vitro* tests [97, 98].

**Fig. 7:**
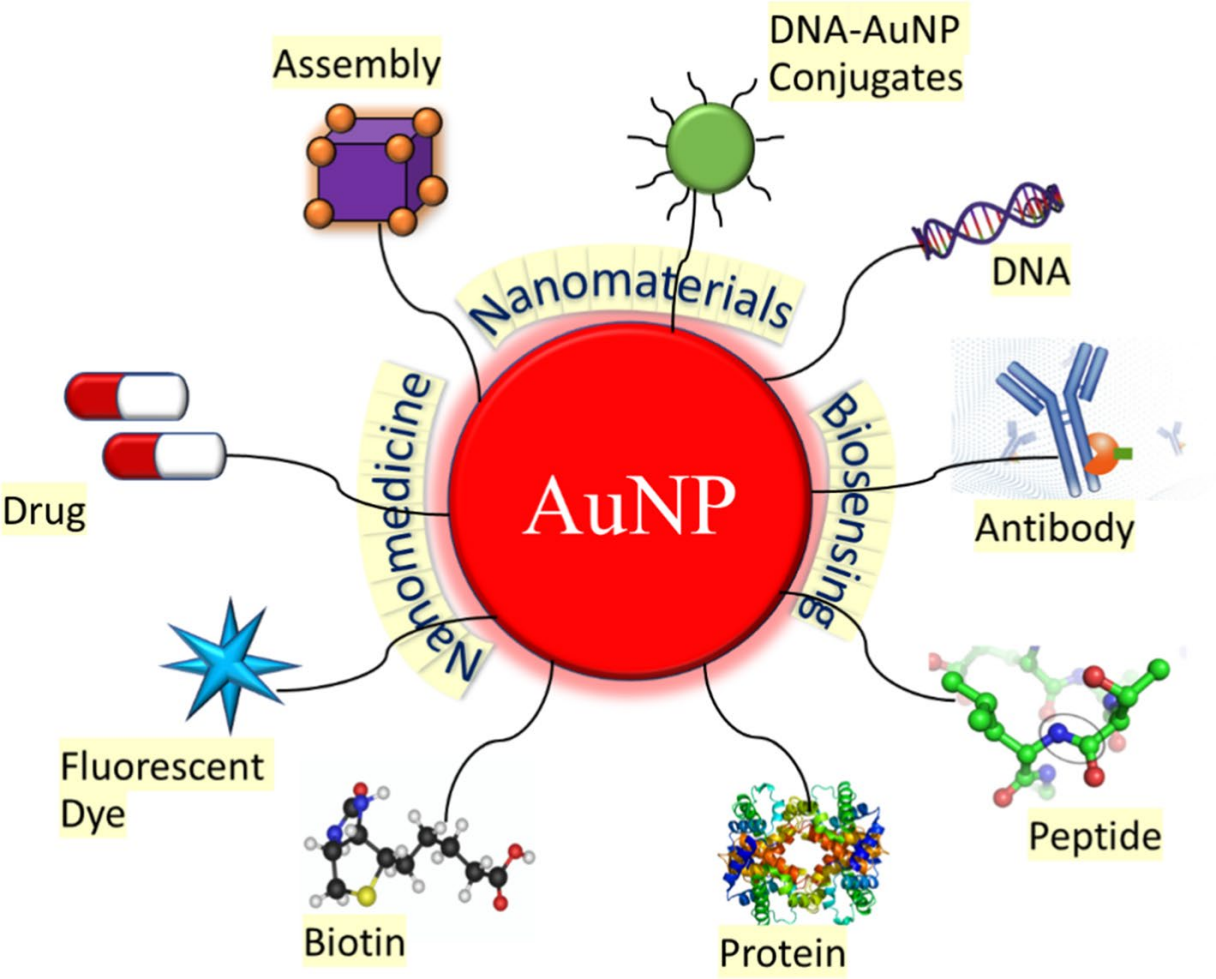
Schematic overview of the possible functionalization and application of gold nanoparticles as nano-carriers for theranostics

Under an external magnetic field, local field inhomogeneity is increased by AuNPs with anisotropic geometries and morphologies. Magnetic imaging was successfully directed by the glutathione (GSH)-responsive Au nanowreath (AuNW). Excessively tiny magnetic iron oxide nanoparticles (ESMIONs) painted on AuNWs effectively quenched with T1-weighted-MRI. After intravenous delivery, the T1 signal of magnetic AuNWs changed from an initial "OFF" state to "ON" due to the significantly higher GSH content in the tumor microenvironment. Higher MRI contrast imaging was possible due to the larger ES-MION assemblies, which improved the tumor accumulation compared to ES-MIONs alone [56, 99]. The possible functionalization of AuNPs as nano-carriers for drug loading which enhances the surface capability for the possible tumor selective accumulation in comparison with free drugs is shown in Fig. 7.

The dysregulated gene expression of tumor cells can be changed during gene therapy to treat cancer. DNA/RNA must be compacted, quickly taken up by cells and released from endosomes, shielded from cytoplasmic and bloodstream breakdown, and delivered to the nucleus efficiently for gene expression to be successfully controlled. To suppress gene expression in a specific fraction of cells, scientists coated 40 nm gold nano-shells with siRNA and Tat peptide-lipid cell internalizing agents. siRNA (short interfering RNA) is a type of RNA molecule that can be used to silence specific genes by binding to complementary messenger RNA (mRNA) molecules and preventing them from being translated into proteins. siRNA is a short peptide sequence called Tat (trans-activating transcriptional activator) that is derived from the human immunodeficiency virus (HIV) and is known to penetrate cell membranes. The Tat peptide is chemically linked to a lipid molecule that allows it to form complexes with siRNA molecules and deliver them into cells. They demonstrated how light-induced siRNA release from a pulsed NIR laser may be used to control the timing and location of gene silencing [100, 101]. Table 2 below compiles few drugs loaded AuNPs with their effect on target cancer cell lines.

**Table 2:** Drugs loaded AuNPs with their effect on targeted cancer cell lines

Cell line targets	Loaded drug	Type of Au nano formulation	Effect	References
MDA-MB-231 (breast cancer), A375 cells (skin cancer cell line)	Doxorubicin	Nanoparticle modified with folate, drug doxorubicin and polyethylene glycol	Enhanced drug delivery, improved anti-tumor activity	[102, 103]
A431 cells skin cancer cells	Curcumin	Nanofibers with Polyvinyl alcohol and Polycaprolactone	Non-toxic biocompatible polymeric scaffold with anti-cancer activity	[104, 105]
Skin (A431), liver (HepG2), and lungs (A549)	Curcumin	2–40 nm nanoparticles	Improves aqueous phase solubility and cellular uptake	[106]
U87 cells, Glioblastoma multiforme (aggressive brain tumor)	Temozolomide	Nanoparticles associated with low-intensity ultrasound	Drug sensitivity enhancement	[107]
HCT116 cells (colorectal carcinoma cell)	Oxaliplatin	Oxaliplatin immuno-nanoparticles (Co-Ox-AuNPs)	Conjugating with anti-DR5 antibody for improved anti-cancer activity& cellular uptake and enhanced site specificity	[108]
A549 (lung epithelial cancer cell line), HCT116, HCT15, HT29, and RKO (colon cancer cell lines)	Oxaliplatin	Platinum-tethered gold nanoparticles	Better cytotoxicity than oxaliplatin alone	[95]
MCF-7 cells	Tamoxifen	Thiol-PEGylated plasmonic gold nanoparticles	Selectively targeting to estrogen receptor positive breast cancer cells with up to 2.7-fold enhanced drug potency	[109]
SKOV3 cells (epithelial cell like human ovarian cancer cell line)	Cisplatin	Synergistic effects of AuNP mediated mild hyperthermia (MHT; 42–43 °C) and cisplatin	Cisplatin mediated cytotoxicity enhanced by 80% with enhanced apoptosis and reduction in tumor volume	[110]
NHI-H69, BEAS-B2 cell lines	Etoposide	Functionalized gold nanoparticles combination of HPMC-E5 and PVA (1:1),	Effective multiplexed therapeutic agent. improved anti-tumor efficacy, with higher loading and release efficiency at physiological pH	[111]
A-549 (Lung) and colon HTC-116 (Colon)	Methotrexate	AuNP–MTX conjugate nanocarrier	Enhancing the therapeutic effect by inducing apoptosis and decreasing the effective doses by half	[112]
MCF7 (breast cancer)	Paclitaxel	PEG coated nanoparticles showed increased maximum drug loading under acidic pH at 24 h incubation period	Increased cytotoxicity (upto 55%)	[113]
HL-7702, Hela, SMMC-7721, and HCT-116 cells	Paclitaxel	Paclitaxel loaded on folic acid and (PEG)-modified AuNPs	Good target selectivity for folate-receptor to induce apoptosis	[114]

##### AuNPs mediated therapy photothermal therapy (PTT) and photodynamic therapy (PDT)

AuNPs are used extensively in photothermal therapy (PTT), which is a key component of cancer treatment. In order to kill cancer cells, gold nanoparticles absorb input photons and turn them into heat and destroys tumor cells by causing biomolecule denaturation and cellular membrane disintegration. [115]. Tumors are more vulnerable to hyperthermia than healthy tissues because of the abnormal vascular anatomy, which is ineffective at dissipating heat.

Near Infra-red (NIR) can directly kill cancer cells by PTT because it is a light absorbent with low toxicity on skin and deep tissue penetration (1 cm deep). Most bio tissue components, such as water, hemoglobin, skin, and other pigments, exhibit minimal absorption and scattering of light in the NIR, allowing the NIR light to penetrate tissues at its greatest depth. Scientists added chemo toxic cisplatin, biocompatible polypeptide poly-L-glutamic acid (PGA), and tumor-targeting folic acid to Au nanorods to make them more useful. The resulting hybrid nanoparticles were able to significantly slow tumor growth and the spread of cancer cells from the primary site to the lung when given systemically to tumor-bearing mice and combined with a localized NIR laser. This was accomplished by destroying the peripheral tumor blood vessels [116].

PDT is a minimally invasive treatment that is often used to treat cancers of the skin, lungs, bladder, esophagus and that of prostate cancer and brain tumors. PDT is typically used to treat early-stage cancers or to alleviate symptoms of advanced-stage cancers. It treats very specific areas of the body, which reduces the risk of damage to healthy tissues. It is also a relatively quick procedure and does not require an extended hospital stay.

PDT generates reactive oxygen species (ROS) from photosensitizers (PS). ROS causes cell death through apoptosis of cancer cells within a 20 nm radius. PS is a drug that is activated by light of specific wavelength (600–680 nm) producing ROS that destroys nearby cancer cells.

During the treatment, the PS is administered to the patient either through injection or orally, depending on the type of cancer being treated. After a specific period of time, the area to be treated is exposed to a specific wavelength of light. This activates the photosensitizing agent, which then destroys the cancer cells by producing a type of oxygen that is toxic to the cells. PDT offers the benefits of excellent tolerance, recurrent usage at the same location, and less invasive surgery. To increase the therapeutic effectiveness, these substances are conjugated to MNPs and exposed to 670 nm laser source, PDT is performed using SPIONs that have been conjugated to pheophorbide-A (a fluorescent photosensitizing agent) [96].

However, there are some risks associated with PDT, such as sensitivity to light, skin burns, and damage to nearby healthy tissues. The photosensitizing agent can also cause some side effects, such as nausea, vomiting, and skin sensitivity.

Using a synergistic effect, PDT/PTT dual-modality therapy, researchers created the GNR-AlPcS4 gold nanorods-photosensitizer complex. In comparison with free AlPcS4-treated cells, GNR-AlPcS4-treated cells displayed a fourfold higher intracellular uptake and improved therapeutic efficiency. PDT and PTT effects can be generated after intravenous injection of GNR-AlPcS4 by individually irradiating with 670 and 810 nm lasers. The tumor growth inhibition for PDT/PTT treatment increased from 79 to 95% when compared to PDT alone [117–120].

#### Magnetic nanoparticles (MNPs)

MNPs have a high magnetic sensitivity and a quick response to an applied external field when they are in the superparamagnetic state and are regarded as the most efficient method for biomedical applications such as MRI, magnetic hyperthermia, tissue repair, immunoassay, detoxification of biological fluids, and cell separation. Cancer theranostics use magnetic nanoparticles made of iron, cobalt, and nickel or combination of cobalt–platinum NPs, iron-platinum NPs, manganese, nickel, or cobalt-ferrite NPs, magnetite or maghemite NPs in spheres, rods, discs, wires, cubes, triangles, polyhedrons, gels, cages and many more shapes. An ideal MNP for clinical applications should be precisely engineered to be stable to act as tracers or deliver drugs to the targeted sites, release drug components only at the targeted sites and have minimal health risks [121].

MNPs can be displayed in several different ways, including as single-phase particles, core–shell NPs with two phases and polymer coatings, multiple cores NPs, or aligned chain arrays that supports the targeting and therapeutic moieties. The surface functionalization and coating of MNPs improve colloidal stability, enable covalent or electrostatic binding of therapeutic cargo, targeting molecules, and/or advanced imaging probes, and thus are crucial in adjusting MNP characteristics like pharmacokinetics, systemic toxicity and clearance rate, non-specific protein adsorption or cell interactions, sustained drug release, among others. The optimal dimensions for intravenous administration of a magnetic nanoparticle system are shown to be between the range of 10–100 nm for pharmacokinetic purposes, as nanoparticles below 10 nm are immediately eliminated via renal excretion while those over 200 nm are rapidly removed from the bloodstream. Carbamate disulfide bridged doxorubicin dimeric prodrug as photothermal agent, and biocompatible hydroxyethyl starch-folic acid conjugates as amphiphilic surfactant to prepare a theranostic nanomedicine to target at TNBC 4T1 tumor tissues has been reported by Wang et al. [122]. Kim et al. [123] reported the use of multifunctional hyaluronate nanoparticle hybrid systems for diagnostic, and theranostic applications as it can enable long-term and efficient delivery of nanoparticles to target sites as well as physiological stabilization of nanoparticles by forming hydrophilic shells.

The surface determines the effectiveness of magnetic nanoparticles for imaging and drug delivery because it affects targeting, cellular uptake, and intercellular localization. Surface charge can be measured by electrophoretic light scattering (ELS) to determine the zeta potential. MNPs are the best for *in vivo* cancer imaging and diagnosis in humans. Superparamagnetic properties allow them to alter the spin–spin relaxation times of nearby water molecules, which allows them to track gene expression, identify tumors, and detect other disorders.

##### Magnetic hyperthermia (MHT)

**Fig. 8:**
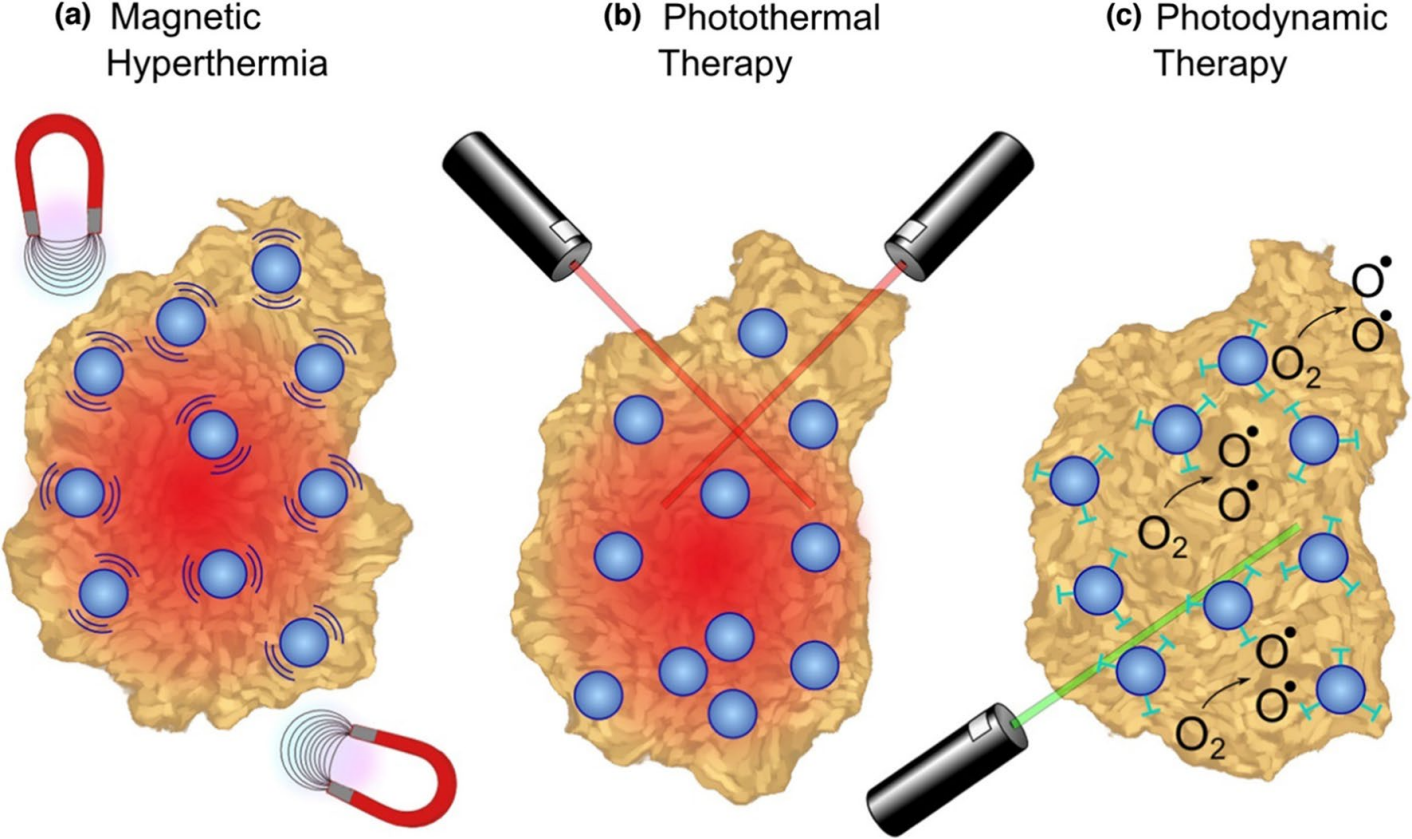
Tumor ablation therapies with Fe_3_O_4_ NPs: **a** In magnetic hyperthermia, an alternating magnetic field causes iron oxide NPs to generate heat, inducing tumor necrosis. **b** In photothermal ablation, light absorbed by NPs is converted to thermal energy causing cell death in the vicinity. **c** For photodynamic therapy, photosensitizing agents attached to NPs are activated by an external light source to create singlet oxygen species that are cytotoxic to cells. Reprinted from [21] under Creative Commons CC-BY-NC-ND

MHT's potential for use in medicine has sparked a lot of curiosity in recent years. The electromagnetic energy of MNPs can be transformed into heat due to which they can cause apoptosis [124]. Magnetite and maghemite are the MNPs that are most frequently employed in cancer treatments [125, 126]. Magnetically separated MNPs increases the efficacy of hyperthermia. The effectiveness of chemotherapy medicines may generally be slightly improved by MHT. For instance, the therapeutic effects of MHT on breast cancer could be significantly improved by combining SPIONs functionalized with multivalent pseudopeptide for delivery of DOX (doxorubicin-drug) [127].Additionally, Pt-Fe-hydroxyapatite MNPs with dual functions were created to treat lung cancer using chemo-hyperthermia [128]. Use of Fe_3_O_4_ NPs in magnetic hyperthermia, photothermal therapy, and photodynamic therapy for cancer treatment is shown in Fig. 8.

### Polymer-based theranostic agents

Polymeric nanoparticles (NPs) can function as nano-carriers and transport various fluorescent dyes or photosensitizers for both therapeutic and photoimaging purposes. Polymer conjugated complexes, microspheres, micelles, and dendrimers have been produced to help in medication delivery to malignant locations and have demonstrated tremendous efficacy against several forms of cancer. The coupling of bioactive compounds to the polymeric matrix enables accurate drug loading and regulation over release kinetics [129]. Polymers such as polyethylene glycol (PEG), poly(D,L-lactic acid), poly(D,L-glycolic acid), poly(ε-caprolactone), chitosan and dextran have already been approved for clinical use in macro formulations. Polymeric nanoparticle show enhanced drug efficacy compared with free drugs via improved drug encapsulation and delivery, prolonged circulation half-life, and sustained or triggered drug release [75]. Peng et al. proposed an activated nanoparticle delivery system for prostate cancers that included stable iron oxide nanoparticles, poly (D,L-lactide-co-glycolide; PLGA), polyethylene glycol-encapsulated epigallocatechin gallate (EGCG), and fucoidan/hyaluronic acid nanoparticles. The system demonstrated precise and targeted nanotheranostics that dramatically reduced the development of orthotopic prostate tumors through a CT based in vivo imaging system [130].

**Fig. 9:**
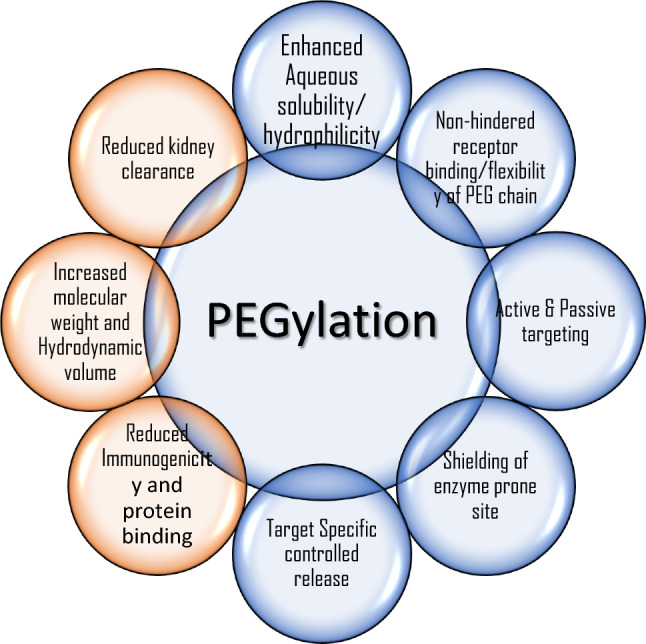
Various outcomes of PEGylation, or modification of therapeutic molecules by PEG conjugation

As shown in Fig. 9, PEGylation (conjugation with PEG), that amend the pharmacokinetic and pharmacodynamic outcomes of therapeutics by decreasing the uptake by reticuloendothelial system, prolonged blood residence, decreased degradation by metabolic enzymes and reduced protein immunogenicity [131]. Islam et al. carried out a study on doxorubicin biodistribution, characterization of the tumor immunologic milieu, cellular doxorubicin uptake, and tumor growth studies were performed in Balb/c mice bearing subcutaneously implanted WEHI-164 fibrosarcoma cells treated intravenously with PEGylated liposomal alendronate doxorubicin (PLAD), PEGylated liposomal doxorubicin (PLD), free doxorubicin, or vehicle. Studies indicated PLAD delivery resulted in higher level of tumor doxorubicin than other methods [132]. It provides various advantages, including a dependable and low-cost method for surface modification, locations for selective attachment of targeted moieties, and the ability to be tuned to show certain optical or magnetic characteristics [133]. These polymeric therapeutic and diagnostic compounds must include four components: an image processing moiety, a drug, a coat for increased bioactivity and durability, and active components for cell targeting [134]. Examples of drugs that have been incorporated into polymeric compounds include paclitaxel, a chemotherapy agent used to treat cancer, and doxorubicin, another chemotherapy agent. These drugs are typically incorporated into the polymeric matrix using techniques such as covalent bonding or encapsulation. Coating component is designed to protect the drug and allow for sustained release over time, for example, polyethylene glycol (PEG), a biocompatible polymer that is commonly used to increase the half-life of drugs in the bloodstream. Other coatings include chitosan, hyaluronic acid, and polyvinyl alcohol.

Polymeric NPs are growing rapidly as a highly integrated theranostic nanoplatform for diagnosis and detection of cancer. It consists of natural and synthetic polymers. Polymeric NPs are synthesized using synthetic polymers such as polyglutamic acid, polyglycolide, polylactic acid, polyanhydride and natural polymers like chitosan, gelatin, sodium alginate, and albumin. The formed polymeric nanoparticles can be obtained as nano capsules, nanospheres, polymeric micelles, drug-polymer conjugates, dendrimers, polymersomes and polyplexes [135]. Polymers approved for use in macro formulation in clinical trials are polyethylene glycol, poly(DL-Lactic acid), poly(DL-Glycolic acid) and poly(caprolactone). Polymeric formulations can thus be loaded with a wide range of therapeutic and imaging agents.

Polymeric NPs provide numerous advantages for cancer therapy, they improve potency over free medicines by improving drug encapsulation and distribution, extending circulation half-life, and allowing for continuous or triggered drug release. Residence time and stability inside the biological system increases when polymeric NPs are treated with successful ways to reduce immunogenicity and antigenicity. Modifying nano-carriers by treating it with PEG reduces immunogenicity and antigenicity as it acts as a barrier for the polymeric nanocarrier, preventing it from being destroyed by stearic hindrance as well as renal clearance. At specific disease site polymer NPs accumulates there either actively by the insertion of targeting moieties specific for a receptor or surface ligand at the region of interest or passively via increased permeability and retention effect. Polymer NPs are highly bio compatible, biodegradable and shows structural versatility [129].

Polymeric NPs have been demonstrated to be efficient carriers of MRI agents such as super paramagnetic iron oxide (SPIOs) and gadolinium (Gd) based compounds. Ye et al. [136] coupled gadolinium (Gd-DO3A) to poly (L-glutamic acid) (PGA) side chains and separated the polymer (Gd-DO3A) into 3 groups with molecular weight of 87, 50 and 28 kilo Dalton (kD). Strong MRI signal amplification was found in the tumor periphery in mice bearing MDA-MB-231 human breast cancer xenografts. The investigations demonstrated that conjugates with higher molecular weights (87 and 50 kD) had longer blood circulation and higher tumor accumulation. Because of their good thermodynamic association constants and low dissociation kinetic rates, polymeric gadolinium NPs offer significant potential in cancer diagnosis in MRI [137].

The use of polymers-based NPs formulations as radionuclide imaging carriers has been investigated. Wide range of copolymers like N-(2-Hydroxypropyl) methacrylamide (HPMA) has been extensively explored by conjugating radionuclide compounds such as ^64^Cu, ^99m^Tc, ^18^F, ^11^C, ^76^Br, ^90^Y, ^111^In construct strong nano-sized delivery systems. The potential of incorporating PET imaging agents into polymer-based nano-carriers, notably for non-invasive viewing and quantification of angiogenic biomarkers has been realized to construct viable nano theranostic platforms. Chen et al. used a PEG linker to connect c(RGDyK), a cyclic RGD peptide ^64^Cu-DOTA and the resulting conjugates demonstrated excellent targeting in brain tumor models [138]. Furthermore, the PEG molecules used to functionalize SPIOs enable additional sites to bind tumor targeting legends like c(RGDfC) which can increase the specificity and efficacy of SPIO-based drug delivery to tumors. Peptide and PET emerging agents like ^64^Cu chelators, which is viable technique for combining drug delivery to tumors with PET/MRI dual-modality imaging. This approach can provide both anatomical and functional information about the tumor, allowing for better visualization and monitoring of drug delivery and treatment response. The detailed description of different type of polymeric NPs is laid in subheads below:

#### Biodegradable polymeric NPs

The biodegradable polymeric NPs are submicron-sized particles containing therapeutic molecules that have been adsorbed, diffused, entangled, connected, or encapsulated into the nanoparticles or a gene of interest contained within a polymeric matrix. Biodegradable polymers, such as poly(lactic-co-glycolic acid) (PLGA), poly(caprolactone) (PCL), chitosan, or gelatin, are chosen for their ability to break down into non-toxic byproducts in the body. These polymers have been extensively used in drug delivery due to their biocompatibility and tunable degradation rates. These polymers are engineered to encapsulate therapeutic agents, such as chemotherapy drugs, small molecules, proteins, or nucleic acids. The encapsulation protects the drug during circulation and allows controlled release at the target site, enhancing the therapeutic effect while minimizing systemic exposure. These nanoparticles are simple to make, relatively inexpensive, biocompatible, non-immunogenic, non-toxic, and water-soluble. These are also valuable as an effective technique of delivering medications to specific tissues/organs, as a method of DNA gene therapy, and for their ability to distribute proteins, peptides, and genes by oral ingestion. Anti-cancer drugs, 5-fluorouracil, dexamethasone, cisplatin, doxorubicin, paclitaxel, triptorelin, xanthone, and others have been shown to be effective when combined with poly (glycolic acid) (PLGA) [139]. After their role is fulfilled, the biodegradable polymer nanoparticles undergo gradual degradation in the body. As they break down, they release their components, which are then metabolized and cleared through normal physiological pathways.

#### Polymeric super magnetic NPs

Super magnetic polymer-based nanotheranostic agents combine magnetic properties with therapeutic and diagnostic functions for applications in disease imaging and therapy. These NPs are utilized to improve the therapeutic presentation of medications and reduce the negative effects associated with conventional cancer treatment. These agents are engineered to have both magnetic and photoacoustic properties, enabling enhanced imaging capabilities and targeted drug delivery. With the use of external magnetism, they may be steered toward to the tumors by magnetic targeting. These NPs could be employed as medication carriers by combining them with synthetic and natural polymers. For drug delivery, polymeric superparamagnetic iron oxide NPs (SPIONs) were combined with polymersomes and micelles [140]. This is attributed to their high drug loading, biocompatibility, stability, fine particle size distribution, prolonged blood circulation, hyperthermia therapy, and controlled drug release. The super magnetic polymer-based agents also incorporate photoacoustic imaging contrast agents, such as organic dyes or other optically absorbing materials which when exposed to laser light generate signals, enabling real-time imaging of the tissue and the distribution of the nanoparticles.

#### Polymeric micelles

A micelle is a collection of surfactant molecules both with hydrophobic and hydrophilic components that self-assemble in an aqueous environment to form arrangements with a hydrophobic focus and a stable hydrophilic encapsulation [141]. The advantages of polymeric micelles include their drug-carrying properties, including the solubilization of insoluble medicines, and precise drug discharge. Polymeric micelles have attractive qualities such as simplicity of micelle synthesis via self-assembly of amphiphilic block copolymer moieties and drug encapsulation via physical entrapment instead of chemical conjugation. These are collected using various techniques (outward from the core), such as preparation of the monomer (G) in the main core or convergent (inner core) procedures. Micelles are effective colloidal transporters of hydrophobic medicines in chemotherapy. The small size of micelles makes them ideal for entering cancer tissue and releasing drugs. Paclitaxel loaded mPEG-PLA polymeric micelle is used for breast cancer treatment [142]. Epirubicin loaded PEG-b-PAH polymeric micelle is used to treat solid tumors [143]. Doxorubicin loaded Pluronic L61 and F127 polymeric micelle is used to target lung cancer[144]. Polymeric micelle of Camptothecin (CPT) is a topoisomerase I inhibitor, is used to kill renal and liver cancer cells [145].

#### Polymer AuNPs

Because of their ease of surface modification, AuNPs coagulated with polymers are good candidates for drug release. AuNPs come in a variety of shapes, including nanorods, nano-shells, nanospheres, nano-stars, nanoboxes, nanocrystals, nanocages, nanoclusters, nanocubes, and triangular bipyramids [146]. Polymer coated AuNPs act as good candidates for drug delivery, with extended circulation time, greater drug loading capacity and stability, and lower cytotoxicity. Chemotherapeutic anti-cancer medications are loaded onto these nanoparticles via covalent and ionic bonding, as well as physical sorption. Table 3 below describes few polymer coated drug-loaded AuNPs used against different cancer cell lines.

**Table 3:** Polymer coated AuNPs as drug delivery vehicles for various cancer cell lines

AuNP modifications	Loaded drugs	Modes of functionalization	Cell lines and models	References
PEG-folate	Curcumin	Hyaluronic acid (HA)-curcumin linking	HeLa cells, C6 glioma cells and Caco 2 cells	[147]
PEG	Paclitaxel	Paclitaxel-PEG-SH linking	HepG2 cells	[148]
PEG-SH	Oxaliplatin	PEG-SH linking	A549, HCT116, HCT15, HT29, RKO	[95]
Folate adapted PEG	Doxorubicin	Physical sorption	KB cells	[149]
Folic acid & PEG-SH	Cisplatin	PEG-SH linking	OVCAR-5, OV-167, HUVEC, OSE	[150]

### Quantum dots

Quantum dots with sizes ranging from 3 to 10 nm that can be employed in imaging diagnosis due to their high fluorescence and wide emission spectrum (the fluorescence spectra vary on their size), particularly in the near infrared (NIR) region (wavelength > 700 nm). Quantum dots surfaces can be changed to improve their stability and biocompatibility, as well as their solubility and functionalization to provide higher specificity for targets [151].

Because of their small size, quantum dots can be used into a wide range of applications, including fluid mixtures, textures, and polymer frameworks. Surface modifications can also aid to prevent unfocused binding, accumulation, and aggregation, making it easier to achieve a clear objective image and delivery [152]. Diverse constraints should be regarded, and a few built-up approaches may be chosen in designing the primary quantum dots for malignant growth analysis and focus on conveyance (disease therapy). Quantum dots were employed in several studies to create *in vitro* fluorescent pictures of human malignant cells from malignancies such as melanoma, ovarian, breast, pancreatic, glioblastoma, ovarian epidermoid, lung, hepatocellular, and adenocarcinoma.

**Fig. 10:**
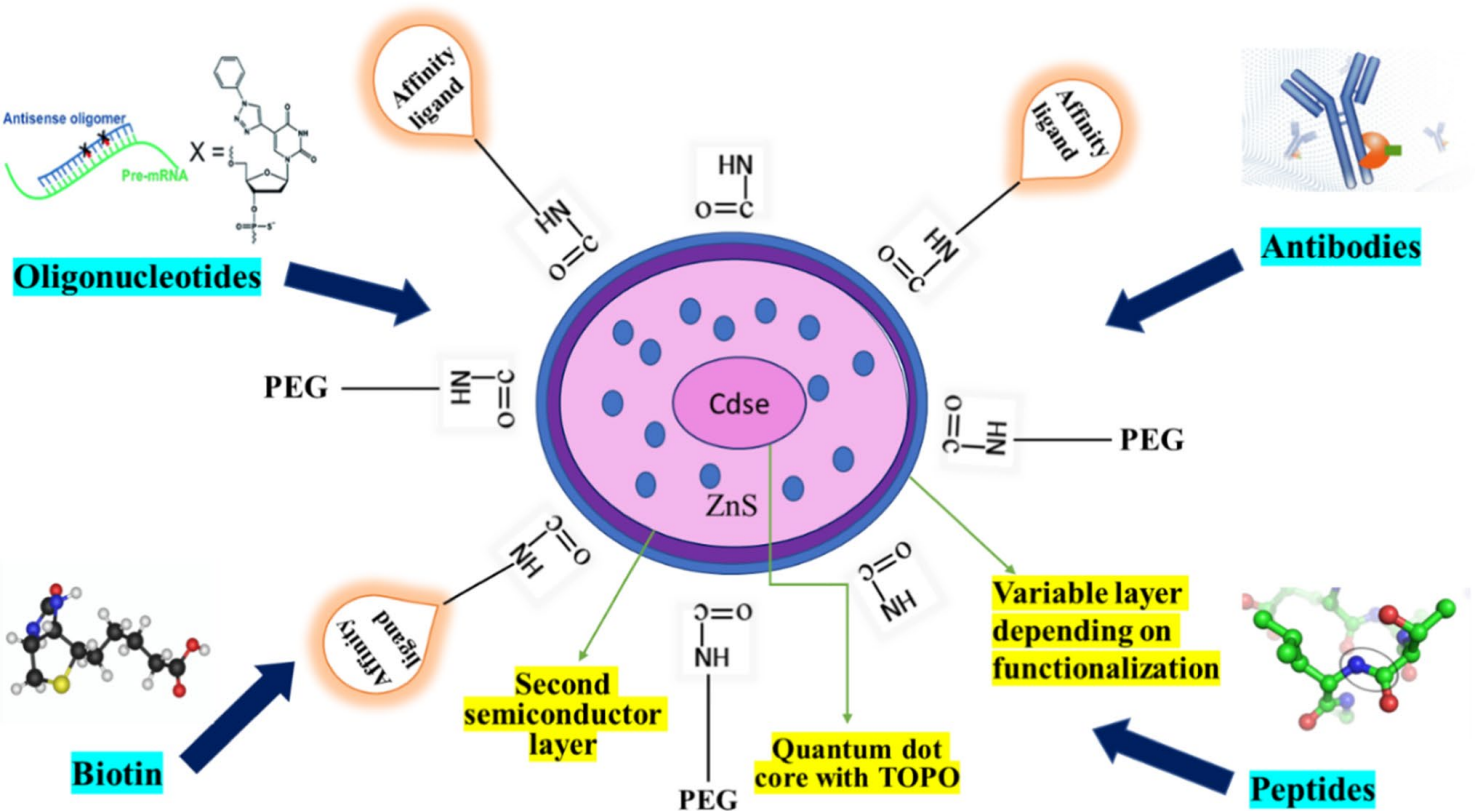
Illustration of Quantum dot assembly of CdSe/ZnS with polymer encapsulation and possible conjugations

Quantum dots are an excellent tool for tumor vascular imaging as well as multi-modal atomic imaging of angiogenesis. Figure 10 depicts the use of CdSe/ZnS quantum dots coated with PEG polymeric interface having affinity with antibodies, peptides, biotin and oligonucleotides. CdSe/ZnS quantum dots (QDs) high fluorescence intensity, long-term stability, and tunable size-dependent emission spectra. CdSe/ZnS QDs were conjugated with folic acid and used for targeted imaging of folate receptor-overexpressing breast cancer cells *in vitro* and *in vivo*. The results showed that the QDs were highly specific and sensitive in detecting cancer cells, with low cytotoxicity [153]. Russo, Maria, et al. functionalized CdSe/ZnS QDs with hyaluronic acid and used for targeted imaging of CD44-overexpressing cancer cells *in vitro* and *in vivo*. The results showed that the QDs could specifically bind to cancer cells and produce bright fluorescence signals, making them a promising tool for cancer diagnosis and treatment [154]. In another study, CdSe/ZnS QDs were conjugated with the peptide RGD (arginine-glycine-aspartic acid) and used for targeted imaging of integrin αvβ3-overexpressing tumor cells *in vitro* and *in vivo*. The results demonstrated that the QDs could specifically target and image tumor cells, with high sensitivity and selectivity [155].

Chung et al. created carbon dots-based fluorescent iron oxide nanoparticles for fluorescence imaging and therapy of cancer cell using microwave irradiation [156]. Ma et al. [157] created a fluorescent probe for detection of protein tyrosine kinase 7 in the peripheral circulation using multi-carbon dots and an aptamer-based signal amplification. Zhang et al. [158], utilized Quantum dots-based fluorophores *in vivo* to detect receptor expression and its downregulation by antibodies. For controlling apoptosis and autophagy in B16F10 epithelial cells and *in vitro* imaging applications, Bajpai et al. [159] produced nitrogen-phosphorous doped carbon dots using a single step heat treatment.

Drug encapsulated nano-carriers targeted at specific organs were modified by conjugating with antibodies, aptamers, folic acid, and others. The use of quantum dots polypeptide nanogels in drug administration expands the possibility of therapy in the detection, imaging, and treatment of malignant growth [160]. Because of their ability to overcome difficulties such as drug resistance, lack of selectivity, and solubility, quantum dots (nanorobots) are genuinely ideal combatants for target-based actions. Various quantum dots, their properties and applications used in cancer imaging are tabulated in Table 4.

**Table 4:** Various quantum dots with properties making them useful for cancer imaging

Quantum Dots	Properties	Applications	References
Duplex metal co-doped CQDs	Synergistic cancer treatment	Reduced tumor development and metastasis	[161]
CQDs—Quinic acid	Biocompatible	Synergic N-CQD, Quinic acid, and Gemcitabine demonstrated enhanced cancer cell cytotoxicity than free Gem	[162]
Black phosphorus QDs	Synergistic chemo-phototherapy and dual-modality cancer imaging	Dual-modality imaging-assisted cooperative therapy improved cancer therapeutic efficacy and patient survival rates. PDT effectiveness increased in a synchronous manner	[163]
Cadmium Selenide (CdSe)	Fluorescent semiconductor nanocrystals, high photoluminescence, good quantum yield, stable emission	Conjugating with antibodies or other targeting molecules, coated with biocompatible materials, such as silica or polymers, to reduce toxicity, imaging agents for cancer	[164]
Indium Phosphide (InP)	High quantum yield, long florescence lifetime, high stability, tunable emission, potential toxicity	Functionalized with targeting molecules, such as antibodies or peptides, to selectively bind to cancer cells and enable imaging of tumors	[165]
Carbon Dots (C-dots)	Biocompatible, low toxicity, good solubility, stable emission, high quantum yield	Have drug delivery capabilities, functionalized with targeting molecules and/or drugs for selectively, used to target specific cancer cells expressing certain biomarkers or antigens	[166, 167]
Copper Indium Sulfide (CuInS_2_)	High stability, tunable emission from red to NIR, good biocompatibility, long lifetimes (100-nano s)	EPR effect to tag tumors, used as multi-modal imaging diagnostic tools, even in drug delivery and phototherapy	[168]
Zinc Oxide (ZnO)	Biocompatible, good quantum yield, stable emission, less toxic, but can generate ROS when exposed to light cause cellular damage and inflammation	Conjugated with targeting agents such as antibodies or peptides to selectively bind to cancer cells, used for image-guided surgery	[169]
Nitrogen, boron/nitrogen, and sulfur-doped graphene quantum dots (GQDs)	Biocompatible multifunctional platforms, water-soluble, show no cytotoxicity, increased chemotherapeutic efficacy	*In vitro* delivery of active agents, multicolor visible/near-IR fluorescence imaging, ratiometric spectral discrimination, and pH-sensing of cancerous environments	[170, 171]
Lead Sulfide (PbS)	High photoluminescence, good quantum yield, good stability, potentially toxic	Useful for *in vivo* imaging of tumors in animal models. Surface coating needed	[172]

## Limitations

Nanotechnology has a significant impact on theranostic because of the production of nanomaterials (1–200 nm) and the identification of unique physical and chemical characteristics that aren't present in their bulk form. Despite all the achievements in nano theranostic there are still many limitations. There is still design, manufacturing, interaction with biomolecules, regulatory, and safety constraints that limit the use of nanotheranostics. These constraints must be considered when designing a viable nano theranostic platform.

Fe_3_O_4_ NPs are thought to be safe as they integrate in the iron pool of the body. However, quantum dots are often coupled with Fe_3_O_4_ NPs as theranostic probes which raises concern about potential side effects. Carbon nanotubes are hazardous as it causes inflammation and cell damage in organs like lungs and liver by creating free radicals which causes oxidative stress by triggering lipid peroxidation. The toxicity of quantum dots is intensified due to specific elements present in them. Size, bioactivity tone, quantum dot fraction, coating materials, shape, and handling constraints are a few factors that affect this danger. Similarly, gold nanoparticles also show reactive oxygen species cytotoxicity as they are non-biodegradable which leads to off-target tissue assembling and toxicity.

The polymeric NPs formulations are frequently polydisperse, making it challenging to fully define them to meet regulatory standards. Furthermore, polymeric nano-carriers that are circulated over an extended period may be harmful or trigger allergic responses. As a result, each novel nanostructure must undergo extensive toxicity testing and analysis. Polymer-based theranostic agents shows stability issue in serums. In lipid based nano agents, concerns have been raised due to their high solubility and quick removal from systemic circulation. Fluorescent imaging cannot determine precise quantitative analyses, accumulation, and pharmacokinetics in deep tissues [173].

Several other negative effects have also been shown to be impacted by different NPs, including cell migration inhibition, decreased pace of wound healing, and endothelial leakage. Aside from these, considerable challenges in the form of bio distribution and pharmacokinetic issues must be addressed. The efficacy of NPs reduces due to the compartmentalization of NPs at the sites of injection as it limits their systemic availability throughout the body. To strike the proper balance in the holistic application, both specificity and bio safety should be given equal attention.

Nanotheranostic platforms are a promising technology for cancer diagnosis and treatment, but they also face several limitations in their design and development. Some of these limitations include:

### Specificity and selectivity

Nanoparticles are frequently utilized in the treatment of cancer because of their unique attributes. They do, however, have drawbacks, such as selectivity and specificity. Their capacity to specifically target cancer cells or tumor locations while avoiding healthy cells is referred to as specificity. This is frequently accomplished by altering the surface of nanoparticles, which enables them to interact with particular receptors found on cancer cells. These alterations may take the form of ligands, antibodies, or peptides that direct nanoparticles to their desired targets. On the other side, selectivity refers to the capacity of the nanoparticles to target cancer cells while preserving healthy ones. This frequently occurs as a result of the particular traits of tumors, such as blood vessel leakage and inadequate lymphatic drainage, which enable nanoparticles to preferentially collect in tumor tissue [174]. Additionally, nanoparticles can be designed to selectively release their therapeutic payload inside the tumor microenvironment, causing the least amount of harm to healthy cells. Achieving this selectivity is challenging, as there are many biological barriers that nanoparticles must overcome to reach cancer cells; hence, molecular targeted therapy is now in focus. The therapeutic index of cancer treatments is improved by the combination of specificity and selectivity. Nanoparticles can deliver a variety of therapeutic agents, such as radiation, gene treatments, and chemotherapeutic medicines, directly to cancer cells, boosting the effectiveness of treatment while reducing adverse effects. When designing and developing nanoparticle-based cancer therapeutics, it is important to consider the heterogeneity of the tumor, alterations in the tumor microenvironment, and potential off-target effects [175].

### Biocompatibility

Nanoparticles should be biocompatible, meaning they do not cause any harm to the human body. Some nanoparticles can cause toxicity or immune reactions, which can limit their clinical application. The biggest issue with nanoparticles is their toxicity, which depends on the specific nanoparticles' form, size, and surface chemistry. Gold nanorods were shown to be more hazardous than spherical particles. The shape and curvature of the nanoparticles also affect how well they circulate in the blood. Nanoparticles may provoke immunological reactions, inflammation, or accumulation in non-targeted locations if they are not adequately biocompatible engineered. When using immune cells to target cancer cells in immunotherapies, biocompatibility is a factor to prevent problems like cytokine storms or autoimmune reactions. Researchers and medical experts are continuously working to create therapies that successfully target cancer cells while limiting damage to healthy tissues through biocompatible design [176, 177]. Biologically derived nanomaterials which are functionalized with as DNA, RNA, aptamers, and antibodies perceive advantages over the nanomaterials produced by conventional methods in terms of economy, ease of production, and reduced toxicity [178].

### Pharmacokinetics

Nanoparticles based drug delivery systems have better pharmacokinetics and are able to navigate the body’s circulatory system and reach the site of the tumor. They should also have a controlled and predictable release of the therapeutic agent. The primary focus should be on examining the process, thermodynamics, and kinetics of adsorption/desorption equilibria for putative drugs on/from carbon nanomaterials with varying purity, surface chemistry, and agglomeration state under various conditions. Major challenges imposed are poor cancer penetration by nanomedicines; the difficulty of estimating the possible effectiveness of an active drug targeting approach for its particular use; the trouble handling nanoformulations that are too complicated in design; the not always advantageous utilization of nanomedicine compositions in medically consistent combination regimens; and less knowledge regarding biological and physiologic principles of tumor progression.

### Manufacturing

Reduced particle size improves the material's overall surface-area-to-volume ratio substantially and changes the surface energy. Manufacturing nanoparticles with consistent quality and quantity can be challenging. There can be batch-to-batch variation, leading to non-reproducibility which can impact their efficacy and safety. Because manufacturing techniques vary, parameters that are employed in other experiments will produce distinct outcomes and must be re-optimized. Optimization and manufacturing of NPs synthesis are difficult and time-consuming processes. The particles are not stable, and they often change in reaction to variations in the environment or over time.

### Cost-effectiveness

Nanotheranostic platforms can be expensive to develop and manufacture. The cost of these platforms can limit their accessibility to patients who may not have the resources to pay for them. The utilization of noble metal nanoparticles (MNPs) in cancer theranostics with unique capability to offer minimally invasive or non-invasive therapeutic approaches. Gold and silver nanoparticles are the primary focus of research due to their favorable attributes such as ease of functionalization, stability, and biocompatibility. Additional noble metallic nanoparticles like platinum (Pt) and palladium (Pd) have been integrated with gold for diverse biomedical applications, particularly in cancer treatment. On the other hand, metals like palladium (Pd), iridium (Ir), rhodium (Rh), osmium (Os), and ruthenium (Ru) are still in the process of catching up in terms of exploration. The limited attention given to these noble metals might be attributed to their scarcity and elevated cost [179].

### Clinical translation

Finally, even if nanotheranostic platforms show promise in preclinical studies, it can be challenging to translate these results to clinical settings as they must be approved by regulatory agencies Getting regulatory approval can be a time-consuming and costly process that requires extensive testing and evaluation. Over the last ten years, there has been a notable rise in research related to the development, composition, and testing of theranostic nanosystems, both in lab settings and in trials with animals and humans. Despite this progress, the FDA has not yet given its approval for the use of these theranostic nanoformulations in clinical applications [180]. One of the most crucial challenges in advancing theranostic nanomedicines from laboratory settings to real-world applications, revolves around establishing the connection between the physical and chemical characteristics of the designed micro- and nanosystems and how they interact with biological systems. Clinical trials can be complex and lengthy, and there is no guarantee that the results will be as promising as in preclinical studies. It is essential to conduct clinical translational studies that assess the effectiveness, potential adverse effects not targeted at the intended site, and the inherent physical and chemical properties of these nanosystems while they are circulating within the body. Therefore, it is essential to conduct clinical translational studies that assess the effectiveness, potential adverse effects not targeted at the intended site, and the inherent physical and chemical properties of these nanosystems while they are circulating within the body. A significant challenge in the clinical advancement of therapeutic developments lies in the lack of clarity surrounding the chemistry, manufacturing, and controls (CMC) aspects, as well as the adherence to good manufacturing practice (GMP) requirements. This becomes particularly pronounced when scaling up the production of intricate nanomedicines, which could introduce additional complexities in terms of CMC and GMP compliance. As a result, there might be a need to refine existing unit operations and implement improvements to meet the standards set by these regulatory frameworks [181].

## Conclusion

Nanotheranostic platforms have tremendous potential for cancer diagnosis and treatment, their development and implementation face several limitations that need to be addressed to ensure their safety, efficacy, accessibility, and regulatory approval. Nanoparticles has been used as a promising candidates for therapeutic agents for cancer therapies like chemotherapy, radiotherapy, phototherapy, gene therapy, and immune therapy for tumor treatment [182]. In this review, several types of nanoparticles such as lipid-based, polymeric-based, inorganic, magnetic carbon-based nanoparticles have been discussed in the appropriate sections above.

In this review article, we describe current advances in the field of nanotheranostics that demonstrate the value of functional nanomaterials in the early detection of cancer and the use of specific drugs to treat it. Researchers are concentrating on designing multifunctional nanotheranostic agents, which include targeted drugs for cancer and photosensitized therapy, for early stage diagnostics and treatment. Due to the intricacy, synergistic effects, unclear mechanisms, and hazardous behavior of these nanoparticles, utilizing nanotheranostic drugs can be challenging. To transform nanotheranostics into clinical trials, several efforts have been made by scientists and researchers. Several nanotheranostic agents, their function in molecular imaging systems, distribution of nanoparticles, targeted drug delivery using imaging techniques, and the mechanism of these nanomaterials' interactions with biological systems have been reviewed. The material employed for the design and fabrication of the nanoplatform and the interaction with the cancer microenvironment are related to the active and passive targeting processes. Non-invasive imaging techniques are therefore crucial in these situations to assess the specificity of receptor binding and internalization processes of the nanosystems within the cancer cells. For such systems, polymeric, metallic, and lipid-based nanotheranostic systems combined with diagnostic and therapeutic functions is required. Nanotheranostics may provide the appropriate medicine at the appropriate dose to the appropriate patient at the appropriate time. These studies could pave the way for the rapid development of effective theranostic nanoplatforms. For it to be successful, pharmaceutical companies must be motivated to conduct clinical trials and continue to introduce novel nanotheranostics into the potential market after necessary approvals.

## Data Availability

Not applicable.
